# Suppression of CSF2RA macrophage polarisation impacts pathological cardiac remodelling in mice

**DOI:** 10.1038/s41598-025-33936-1

**Published:** 2026-01-09

**Authors:** Georgios Kremastiotis, Yong Li, Andrew Bond, Daire Shanahan, Karina Di Gregoli, Alastair W. Poole, Sarah J. George, Jason L. Johnson

**Affiliations:** 1 Laboratory of Cardiovascular Pathology, Bristol Medical School, Faculty of Health Sciences, University of Bristol, Bristol, UK; 2 School of Physiology, Pharmacology & Neuroscience, Faculty of Life Sciences, University of Bristol, Bristol, UK; 3 Laboratory of Cardiovascular Pathology, Translational Health Sciences, Bristol Medical School, Faculty of Health Sciences, University of Bristol, Level 7, Bristol Royal Infirmary, Bristol, BS2 8HW UK; 4 Present Address: National Heart and Lung Institute, Imperial College London, Sir Michael Uren Building, 86 Wood Lane, White City Campus, London, W12 0BZ UK

**Keywords:** Cardio-immunology, CSF2RA, Fibrosis, Inflammation, Macrophages, Cardiology, Cell biology, Diseases, Immunology

## Abstract

Granulocyte-macrophage colony-stimulating factor (GM-CSF; CSF2) has emerged as an important regulator of pro-inflammatory macrophage polarisation, with pro-inflammatory responses recognised to influence cardiac remodelling and repair after cardiac injury. We hypothesised that selective inhibition of CSF2RA, responsible for mediating CSF2 signalling in monocyte/macrophages, could promote reparative responses after cardiac injury through modulating macrophage/fibroblast communication. We demonstrate for the first time that pharmacological CSF2RA inhibition alters the inflammatory response and cardiac remodelling in response to injury, through promoting pro-fibrotic macrophages and beneficial effects upon cardiac fibroblast differentiation, limiting border zone fibrosis, and encouraging scar maturation, culminating in improved cardiac function. Proteomic and in vitro analyses revealed that loss of CSF2-mediated signalling promotes pro-fibrotic macrophages, which facilitate myofibroblast dedifferentiation, in part through decreased CTSZ expression and concomitant augmented CXCL10/CXCR3 signalling. Providing translational potential, we establish that pharmacological CSF2RA inhibition modulates macrophage responses and associated fibroblast function to promote cardiac remodelling, repair, and recovery. These actions may also be applicable to other inflammatory conditions, non-resolving fibrosis, and wound healing.

**Supplementary Information:**

The online version contains supplementary material available at 10.1038/s41598-025-33936-1.

## Introduction

Pathological cardiac remodelling describes the significant structural and functional changes observed during the healing response within the damaged myocardium. Adverse remodelling predisposes patients to heart failure, due to significant left ventricular (LV) dilatation^1^ and therefore ominously increases future risk of hospitalisation and death^2^. Infarct area, associated hypertrophy, and fibrotic scar formation negatively correlate with LV ejection fraction, an indicator of heart contractility and function^3^. Such structural changes after cardiac injury are attributed to cellular and extracellular processes observed during three overlapping stages: the initial inflammatory response, including infiltration of the injured myocardium by monocytes and monocyte-derived macrophages; the proliferative phase, during which cardiac fibroblasts transition to a myofibroblast phenotype and proliferate to orchestrate wound healing and remodelling responses including collagen synthesis; and lastly, the reparative phase, involving rearrangement of deposited collagens and other extracellular matrix (ECM) proteins alongside myofibroblast dedifferentiation to facilitate formation of a stable and mature scar^4,5^. However, it is recognised that a biphasic inflammatory response is mounted after cardiac injury, with infiltration of monocytes giving rise to pro-inflammatory macrophages, which secrete a myriad of cytokines and induce further cell death^6–8^. Successively, macrophages acquire an anti-inflammatory and pro-healing phenotype, which facilitates ECM remodelling and reparative mechanisms^6–8^. Relatedly, pro- and anti-inflammatory macrophages have been proposed to release competing signalling factors into the microenvironment which divergently modulate intercellular communication within the injured myocardium, particularly within the expanding myofibroblast population^9^.

Colony stimulating factors (CSFs) are secreted glycoproteins that function as haematopoietic cell growth factors and differentiate progenitor cells into macrophage (M-CSF), granulocyte (G-CSF) or granulocyte and macrophage (GM-CSF) colonies^10^. GM-CSF (CSF2) and M-CSF (CSF1), dictate monocyte to macrophage differentiation and generate distinct pro-inflammatory and pro-fibrotic macrophage subsets respectively, both in vitro and within atherosclerotic plaques^11,12^. CSF2^13,14^ and CSF1^15,16^ are upregulated in the ischaemic myocardium of animals and patients post-myocardial infarction (MI), supporting the hypothesis that macrophage phenotype and function can be antagonistically regulated by CSFs after cardiac injury and during pathological cardiac remodelling.

CSF2 expression is strongly upregulated in human and mouse cardiac fibroblasts after an insult and triggers bone marrow haematopoiesis alongside promoting neutrophil and monocyte recruitment to the affected myocardium^13,17^. Additionally, increased levels of CSF2 in the plasma from patients with acute MI are observed for up to one month following ischaemic injury and significantly correlates with severe LV dysfunction and heart failure occurrence^14^. Previous findings showed that Csf2rb deletion inhibited neutrophil and monocyte infiltration in the infarcted myocardium and improved cardiac function^13,17^. While neutrophil contribution to early pathological cardiac remodelling is undeniable^18,19^, it is speculated that persisting CSF2 levels at one-month post-injury will influence monocyte-/macrophage-driven effects, as these cells dominate the healing process. Moreover, CSF2-mediated signalling requires a selective receptor (CSF2RA), and while a further common subunit (CSF2RB) is also necessary, this subunit is also essential for IL-3 and IL-5 receptor-mediated signalling, demonstrating that approaches modulating or targeting CSF2RB are not specific to CSF2^20^.

Consequently, we utilised novel pharmacological inhibition of CSF2RA to explore a direct role for CSF2-polarised macrophages in an experimental model of pathological cardiac remodelling. Providing pre-clinical translational insight, we demonstrate that inhibition of CSF2RA permits effective remodelling through facilitating accelerated reparative cardiac fibrosis. Such beneficial effects are afforded through promotion of CSF1-driven anti-inflammatory and pro-fibrotic effects. Subsequent favourable actions were associated with improved cardiac morphology and contractility. Using in vitro and proteomic approaches, we explored the disparate effects of CSF2-directed and CSF1-directed macrophage polarisation on cardiac myofibroblast behaviour, revealing CSF2-polarised macrophages dampen characteristics associated with myofibroblast dedifferentiation, through a cathepsin Z/CXCL10-mediated mechanism.

## Results

### Csf2ra Inhibition promotes reparative cardiac remodelling

**Fig. 1:**
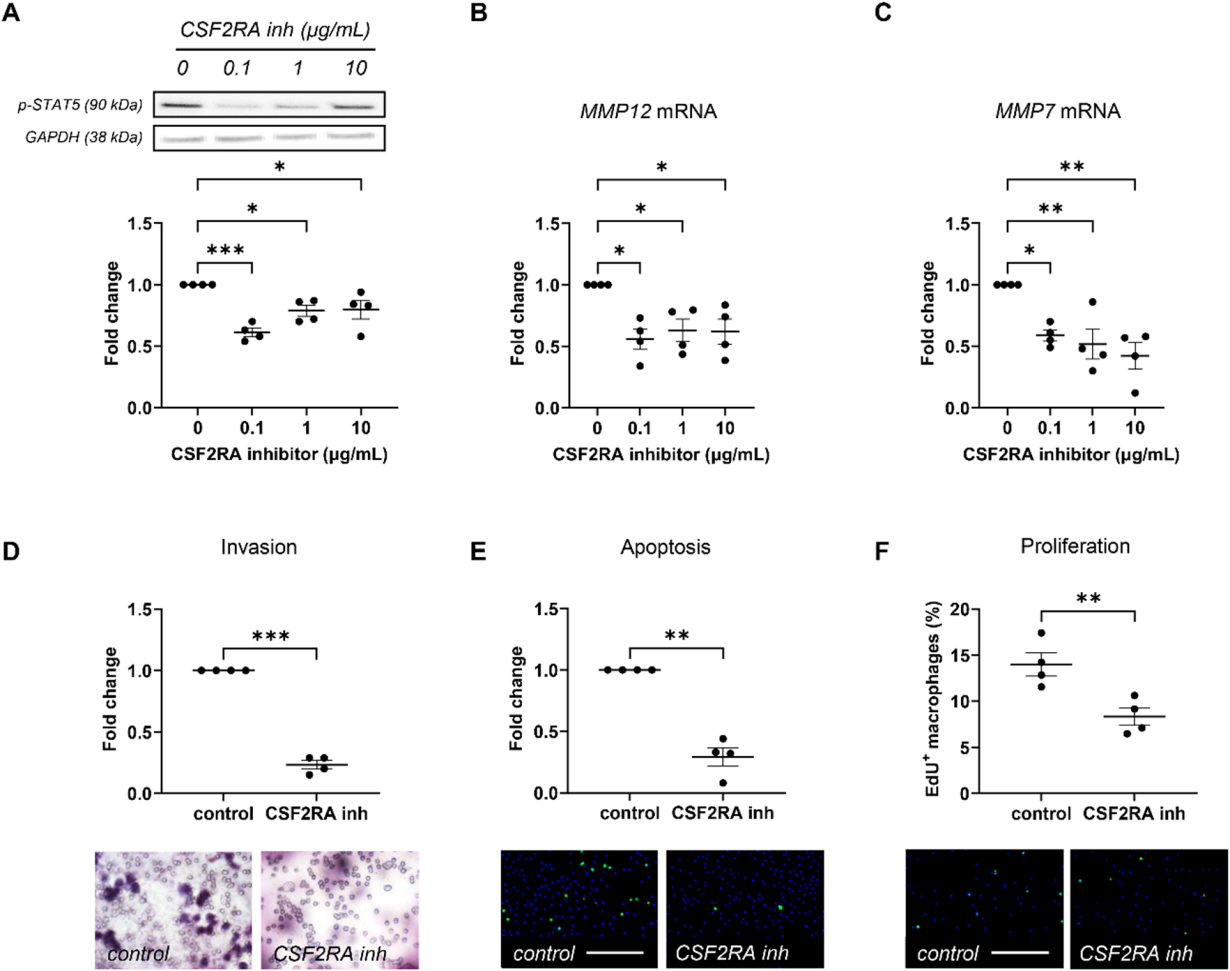
CSF2RA inhibition suppresses CSF2 signalling and associated macrophage behaviour. **(A)** Quantification and representative images of human monocyte-derived macrophages polarised with CSF2 (20 ng/mL) and treated for 4-hours with a CSF2RA peptide inhibitor (E21R) at 0.1, 1, and 10 µg/mL for pSTAT5. Comparative mRNA expression of **(B)** *MMP12* and **(C)** *MMP7* in CSF2 polarised macrophages treated for 24-hours with a CSF2RA peptide inhibitor (E21R) at 0.1, 1, and 10 µg/mL. Quantification and representative images of CSF2-polarised macrophages treated for 24-hours with a CSF2RA peptide inhibitor (E21R) at 0.1 µg/mL for: **(D)** invasion as assessed through invasion within Matrigel-coated transwells, **(E)** FasL-induced apoptosis, as assessed through cleaved caspase-3 immunocytochemistry (green), and **(F)** proliferation, as assessed through EdU incorporation (green). All groups are *n* = 4. Statistical significance is reported as *P < 0.05, **P < 0.01, or ***P < 0.001, using an ordinary one-way ANOVA in panels A-C, and paired Student’s t-test in panels D-F. Scale bar represents 200 μm and is applicable to panels E and F.

We deployed a therapeutic approach and evaluated the potential benefit of CSF2RA inhibition upon cardiac inflammation, reparative remodelling, and function in response to acute myocardial injury. We utilised a peptide inhibitor (E21R) which blocks the action of CSF2 by interacting selectively with the CSF2-specific alpha-chain of the CSF2 selective receptor (CSF2RA)^21^. Validation studies in human monocyte-derived macrophages demonstrated that the CSF2RA inhibitor suppressed CSF2 signalling, as observed through decreased STAT5 phosphorylation and reduced mRNA expression of *MMP12* and *MMP7* (Fig. 1A-C), two STAT5-responsive genes. Furthermore, the selective peptide inhibitor repressed the deleterious characteristics of CSF2-polarised macrophages, such as invasion, susceptibility to apoptosis, and proliferation (Fig. 1D-F).

**Fig. 2:**
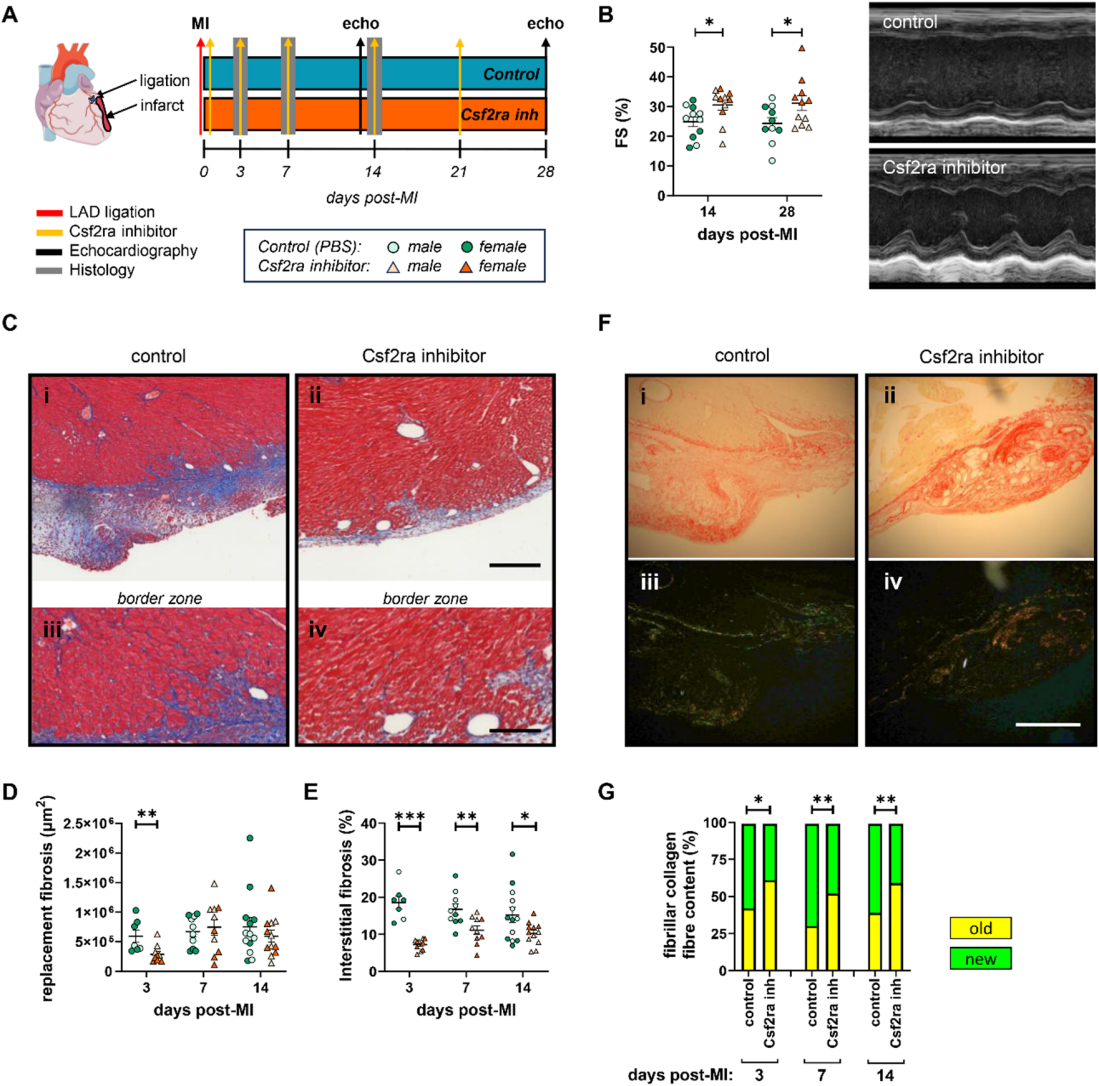
CSF2RA inhibition enhances cardiac function and reduces border zone fibrosis, with concomitant scar collagen maturation post-myocardial injury. **(A)** Experimental schematic of CSF2RA -inhibitor-treated mice subjected to MI. **(B)** Quantification and representative M-mode images of fractional shortening (FS%) at days 14 (control *n* = 11, CSF2RA inhibitor *n* = 11) and 28 post-MI (control *n* = 10, CSF2RA inhibitor *n* = 11). **(C)** Representative images of the infarct and border zone fibrosis from Masson’s-trichrome-stained hearts in control (i, iii) and CSF2RA inhibitor-treated mice (ii, iv). Quantification of **(D)** replacement (µm^2^) and **(E)** interstitial fibrosis (%) (*n* = 7–13). **(F)** Representative images of Picrosirius-Red-stained hearts in control and CSF2RA inhibitor-treated mice, under brightfield (i, ii) or linearly polarised light (iii, iv). **(G)** Quantification of fibrillar collagen fibre content, (*n* = 7–13). Statistical significance is reported as *P < 0.05, **P < 0.01, and ***P < 0.001 using an unpaired Students t-test in Panels B, D, and E, or *P < 0.05 and **P < 0.01 using a Fishers exact test in Panel G. Scale bars represent 200 µM and are applicable to all panels in C and F.

Accordingly, acute myocardial injury was induced in male and female C57Bl/6 N mice through permanent left anterior descending (LAD) coronary artery ligation, followed by intraperitoneal administration of the pharmacological CSF2RA inhibitor (E21R) for up to four weeks, with hearts histologically analysed at 3-, 7-, and 14-days post-injury (Fig. 2A). Administration of the CSF2RA inhibitor improved LV function and structure post-LAD ligation. B-mode scans support the induction of myocardial injury and dysfunction and further highlight the beneficial effect of CSF2RA inhibition (Supplementary Figure S1A). Assessment of fractional shortening (FS) and ejection fraction (EF) from M-mode images revealed a significant improvement in cardiac function at 14-days post-injury, with augmented cardiac function remaining apparent at 28-days post-injury (Fig. 2B, Supplementary Table S1-2). This finding was associated with a significant increase in the LV posterior wall (LVPW) thickness at systole and a marginal increase at diastole at 14-days post-injury, overall suggesting enhanced thickening and contraction of the LV, following CSF2RA inhibition (Supplementary Table S2). Although the average LVPW thickness was comparable between control and CSF2RA inhibitor treated mice at 28-days post-injury, the wall thickness fractional change was significantly increased (*p = 0.0161) following CSF2RA inhibition (*n* = 11; 41.93% ± 5.29), in comparison to control mice (*n* = 9; 22.02% ± 5.26). This finding suggests augmented wall thickening during contraction and an improved ratio of wall thickness at systole to diastole. Lastly, a reduction in the LV internal diameter (LVID) at both systole and diastole was observed at 28-days post-injury (Supplementary Table S2), indicative of LV contraction, while the control mice presented with increased LVID suggesting that the LV chamber remains dilated. Interestingly, we observed a prominent increase in the FS of female mice treated with the CSF2RA inhibitor at 14- (*p* < 0.05) and 28-days post-MI (*p* < 0.01). While the male mice also presented with increased FS, the results did not reach statistical significance, although this may be due to the small group size.

Histologically, CSF2RA inhibition limited initial myocardial injury, as evidenced through reduced injury-associated (replacement) fibrosis 3-days post-injury (Fig. 2Ci-ii, 2D & Supplementary Figure S1B), however this effect did not persist at 7- and 14-days post-injury. In line with the sex effect observed in the FS of the CSF2RA inhibitor-treated mice, the reduced fibrosis at 3-days post-MI was more prominent in female mice (*p* < 0.01) compared to their male counterparts. Given the restricted infarct size in our model, there was no pronounced long-term effects on cardiac remodelling. During the late-stage reparative phase at 28-days post-MI remodelling, we observed no significant differences between the groups or further infarct expansion. The total replacement fibrosis area remained comparable between control (340,561 ± 55,308 µm^2^, *n* = 12) and inhibitor-treated mice (359,010 ± 72,975 µm^2^, *n* = 12). Therefore, we focused all subsequent analyses on the early inflammatory and reparative stages (3-, 7-, and 14-days post-MI).

Regardless, we observed a robust and consistent reduction of interstitial fibrosis in hearts of mice treated with the CSF2RA inhibitor compared to controls (Fig. 2Ciii-iv, 2E). Additionally, CSF2RA inhibition was associated with augmented deposition of old (red) collagen fibres, suggesting accelerated maturation and reparative remodelling at the injury site (Fig. 2F and G & Supplementary Figure S1C). Suppression of the interstitial fibrosis and improved collagen maturation are indicative of a resolving fibrotic response and account for the improvement of the LV systolic function. At a cellular level, a modest increase of granulation tissue at the site of injury was noted at day-3 and a significant increase at day-14 post-injury in the inhibitor-treated mice. (Supplementary Figure S1D), a characteristic associated with long-term improvement of cardiac remodelling and function in rodents^22^.

**Fig. 3:**
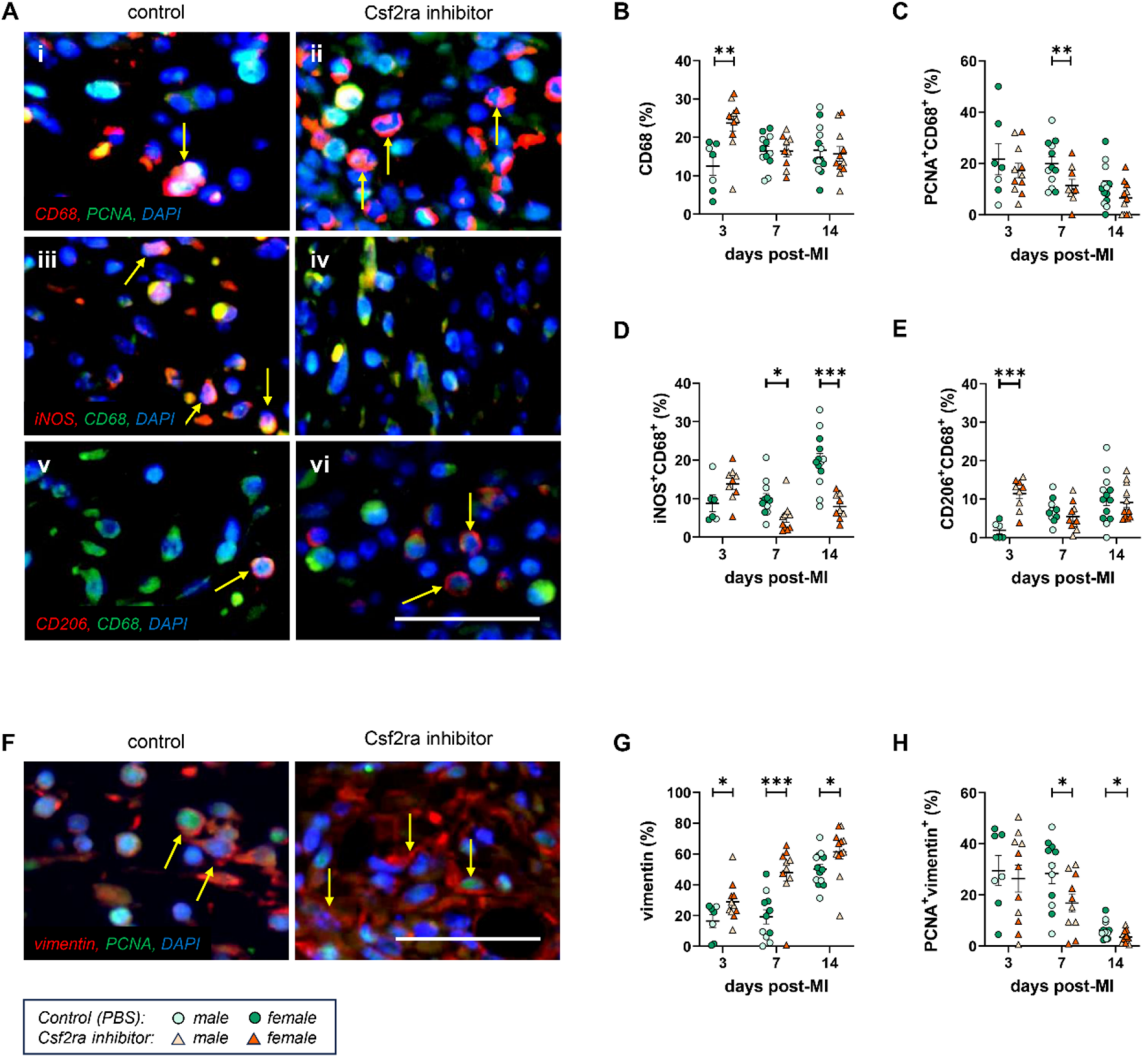
CSF2RA inhibition accelerates anti-inflammatory macrophage and cardiac fibroblast accumulation post-myocardial injury. **(A)** Representative images of dual fluorescence immunohistochemistry for PCNA (green) and CD68 (red) (i, ii), iNOS (red) and CD68 (green) (iii, iv), or CD206 (red) and CD68 (green)(v, vi); in all instances, cells positive for both markers are observed as yellow/white with examples indicated by arrows, with DAPI used as a nuclear dye. Quantification of **(B)** macrophage density (*n* = 7–13), **(C)** proliferation (*n* = 7–13), and **(D)** expression of iNOS (*n* = 6–13) and **(E)** CD206 (*n* = 6–13). **(F)** Representative images of dual fluorescence immunohistochemistry for PCNA (green) and vimentin (red); cells positive for both markers are observed as yellow/white with examples indicated by arrows, with DAPI used as a nuclear dye. Quantification of **(G)** cardiac fibroblast density (*n* = 7–13) and **(H)** proliferation (*n* = 7–13). Statistical significance is reported as *P < 0.05, **P < 0.01 or ***P < 0.001, using unpaired Students t-test. Scale bars represent 200 µM and are applicable to all panels in A and F.

Inhibition of CSF2RA positively altered cellular composition at the site of cardiac injury and remodelling, as demonstrated through heightened accumulation of macrophages 3-days post-injury (Fig. 3Ai-ii, 3B), which was in the main independent of their proliferative capacity (Fig. 3Ai-ii, 3 C). CSF2RA inhibition abrogated macrophages acquiring a pro-inflammatory phenotype, highlighted by a robust reduction of iNOS-positive macrophages within remodelling regions of inhibitor-treated mice at 7- and 14-days post-injury, in comparison to controls (Fig. 3Aiii-iv, 3D). Additionally, CSF2RA inhibition resulted in an accelerated accumulation of CD206-positive macrophages at day 3 post-injury (Fig. 3Av-vi, 3E). CSF2RA inhibition increased cardiac fibroblast accrual (Fig. 3F and G), alongside diminished proliferation rates (Fig. 3H). CSF2RA inhibition also increased the proportion of cardiac fibroblasts displaying a myofibroblast phenotype (α-SMA-positive), especially at earlier timepoints (Supplementary Figure S1E). Moreover, CSF2RA inhibition was associated with a marked increase in angiogenesis (Supplementary Figure S1F), which was not connected with effects on endothelial cell viability (Supplementary Figure S1G). Lastly, the effect of CSF2RA inhibition on circulating biomarkers was evaluated at day 7 post-injury. Multiplex analyses of plasma chemokines and cytokines following CSF2RA inhibition highlighted significant changes in the cytokine/chemokine plasma proteome (Supplementary Table S3). Notably, a network of pro-fibrotic molecules was upregulated (Supplementary Figure S2), including CCL1, CCL3, CCL4, CCL12, and IL-10, with known pertinent beneficial actions in post-MI fibrosis^23,24^.

Collectively, our results indicate that selective CSF2RA inhibition impacted pathological cardiac remodelling and improved cardiac function in response to injury. The effects were mediated in part through scar maturation, restriction of interstitial fibrosis, and due to abrogation of infiltrating macrophages acquiring a pro-inflammatory phenotype. The effects of CSF2RA inhibition were more prominent in female mice, which displayed a robust increase in FS and a reduction in 3-day replacement fibrosis; at a cellular level, these changes were associated with increased accumulation of CD68^+^ macrophages in the infarcts of CSF2RA inhibitor-treated female mice (*p* < 0.01), in comparison to their male counterparts, which followed the same pattern, without reaching statistical significance. Together these findings highlight CSF2RA inhibition as a therapeutic strategy to calibrate cardiac remodelling and restore cardiac function in response to acute myocardial injury.

### CSF2-polarised macrophages sustain a cardiac myofibroblast phenotype, alongside increased migratory and contractile capacity

In agreement with our in vivo observations from the mouse model of cardiac dysfunction and fibrosis, evidence from macrophage pathophysiology in atherosclerotic plaques suggests that macrophages exposed to CSF1 (M-CSF) alone give rise to an anti-inflammatory, pro-fibrotic phenotype, while exposure to CSF2 (GM-CSF) in combination with CSF1 generates a pro-inflammatory subset^12,25–29^. Accordingly, to emulate our in vivo approaches, we assessed the effect of macrophages with and without CSF2-mediated signalling/polarisation (referred to as + CSF2 and -CSF2) upon cardiac fibroblast behaviour, focusing on accepted hallmarks of fibroblast activation^30^.

**Fig. 4:**
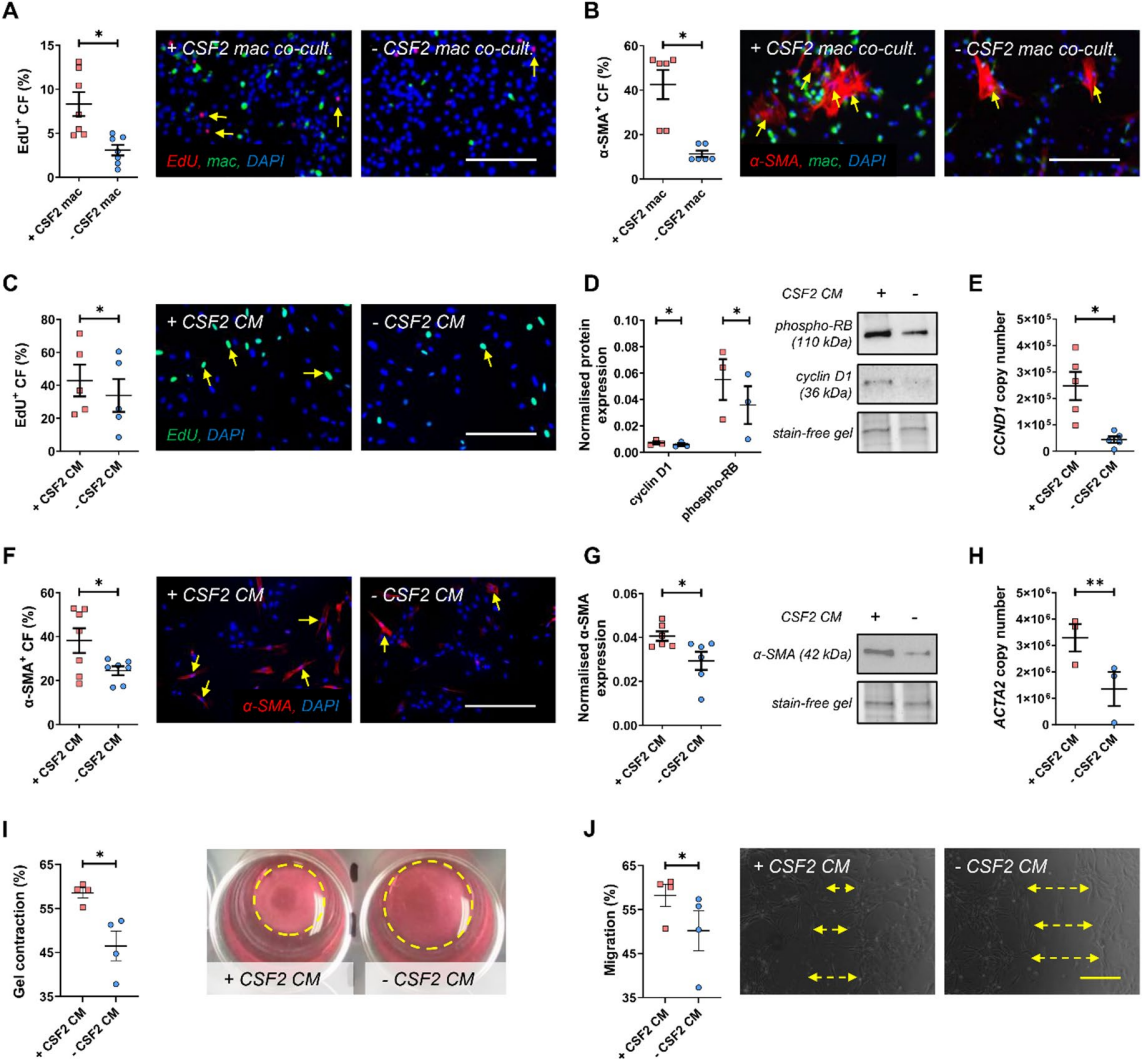
CSF2 polarised macrophages sustain a cardiac myofibroblast phenotype, alongside increased migratory and contractile capacity. Human cardiac fibroblasts and human primary + CSF2 or -CSF2 macrophages (green) were co-cultured for 24 h, and fibroblast activation hallmarks were assessed. Quantification and representative images of **(A)** cardiac fibroblast proliferation, assessed through EdU incorporation (red; *n* = 5); **(B)** activation marker, α-SMA (red; *n* = 6). Human cardiac fibroblasts were treated with macrophage secretome with or without CSF2 polarisation (CM) for 24 h, unless otherwise stated. Quantification and representative images of cardiac fibroblast proliferation, assessed through **(C)** EdU incorporation (green; *n* = 5), **(D)** phospho-RB and cyclin D1 protein expression (*n* = 3), and **(E)** *CCND1* copy number (*n* = 5); activation marker, α-SMA, assessed through **(F)** immunocytochemistry (red; *n* = 7), **(G)** α-SMA protein expression (*n* = 6), and **(H)** *ACTA2* copy number (*n* = 3). **(I)** Cardiac fibroblast contraction was assessed through collagen gel contraction (*n* = 4), and **(J)** migration was assessed through scratch-wound assay for 48 h (*n* = 4). Statistical significance is reported as *P < 0.05 or **P < 0.01, using paired Students t-test. White scale bar represents 200 µM and is applicable to panels A-C and F; yellow scale bar represents 1 mm and is applicable to panel J; panel I shows 24-well plates.

Co-culture of human cardiac fibroblasts with macrophages lacking CSF2-mediated signalling (-CSF2) decreased the proliferative rate of cardiac fibroblasts, as evidenced through the reduced percentage of cells incorporating EdU (Fig. 4A), when compared to CSF2 polarised macrophages (+ CSF2). Similarly, macrophages without CSF2-dependent polarisation decreased cardiac fibroblast α-SMA expression (Fig. 4B), inferring CSF2-directed macrophages impede dedifferentiation of cardiac myofibroblasts and therefore potentially blunt fibrosis resolution^31,32^. As collagen type 1 constitutes the majority of deposited fibrillar collagen within a post-MI scar^33^, we assessed protein expression in our co-cultures; however, no difference was observed by immunocytochemistry within fibroblasts co-cultured alongside macrophages with or without CSF2-mediated signalling (Supplementary Figure S3A). Equally, pro-collagen type 1 expression was not altered in macrophage-cardiac fibroblast co-cultures (Supplementary Figure S3B).

To ascertain whether the disparate effects of + CSF2 and -CSF2-directed polarisation upon cardiac fibroblast behaviour were due to secreted factors, ancillary studies were conducted using the conditioned media (henceforth referred to as the secretome) from macrophages with and without CSF2-directed polarisation. Mirroring the co-culture findings, the secretome from -CSF2 macrophages decreased cardiac fibroblast proliferation (Fig. 4C), with Western blotting of phospho-RB and cyclin D1 proteins (Fig. 4D), alongside qPCR analysis of CCND1 transcript levels (Fig. 4E) confirming the effects upon proliferation. Also paralleling the co-culture results, cardiac fibroblasts exposed to the secretome from -CSF2 macrophages exhibited reduced α-SMA expression, shown through immunocytochemistry (Fig. 4F), and supported by Western blotting (Fig. 4G) and qPCR for the ACTA*2* transcript (Fig. 4H). Assessment of fibrillar collagens at the mRNA level revealed significantly decreased *COL3A1* levels in fibroblasts exposed to the secretome from macrophages lacking CSF2 signalling, whereas *COL1A1* and *COL2A1* did not show consistent changes (Supplementary Figure S3E-G). Accordingly, no differences were identified at the protein level for collagen type 1 (Supplementary Figure S3C) or pro-collagen type 1 (Supplementary Figure S3D), as assessed by immunocytochemistry, alongside Western blotting of conditioned media and cell lysates (Supplementary Figure S3H-I).

Functionally, the secretome from -CSF2 macrophages decreased cardiac fibroblast contractile (Fig. 4I) and migratory capacity (Fig. 4J), in comparison to + CSF2 macrophage secretome, suggesting lack of CSF2 signalling permits myofibroblast dedifferentiation. Indeed, it is accepted that fibroblasts differentiate towards a myofibroblast phenotype in vitro, as a response to high-tension culture surfaces, such as plastic^30,34^. In accord, high α-SMA expression was observed in naïve (baseline) human cardiac fibroblast cultures (Supplementary Figure S4A), supporting their transition to a myofibroblast phenotype in culture. Interestingly, addition of secretome from + CSF2 macrophages had no effect, however, α-SMA levels were diminished by the secretome from -CSF2 macrophages (Supplementary Figure S4A). Accordingly, ancillary experiments were conducted where cardiac fibroblasts were cultured within free-floating collagen gels to mimic a low-tension environment which resulted in reduced fibroblast differentiation towards a myofibroblast phenotype, as evidenced by low α-SMA expression, which was increased through addition of + CSF2 macrophage secretome (Supplementary Figure S4B). Replicating the effects observed on plastic, the secretome from -CSF2 macrophages reduced cardiac fibroblast α-SMA expression, assessed with confocal microscopy of collagen gels (Supplementary Figure S4B) or extraction of cardiac fibroblasts (Supplementary Figure S4C). Lastly, the secretome from -CSF2 macrophages abrogated the contractile capacity of cardiac fibroblasts within free-floating collagen gels (Supplementary Figure S4D). These findings indicate that in the absence of CSF2 signalling, macrophages can induce changes in myofibroblast characteristics that are associated with their dedifferentiation towards a resident fibroblast form, in vitro, with the results consistent across high- and low-tension environments.

### Loss of CSF2 macrophage signalling promotes CXCL10 expression and CXCR3-induced cardiac fibroblast dedifferentiation, while CSF2-macrophage-derived CTSZ degrades CXCL10

**Fig. 5:**
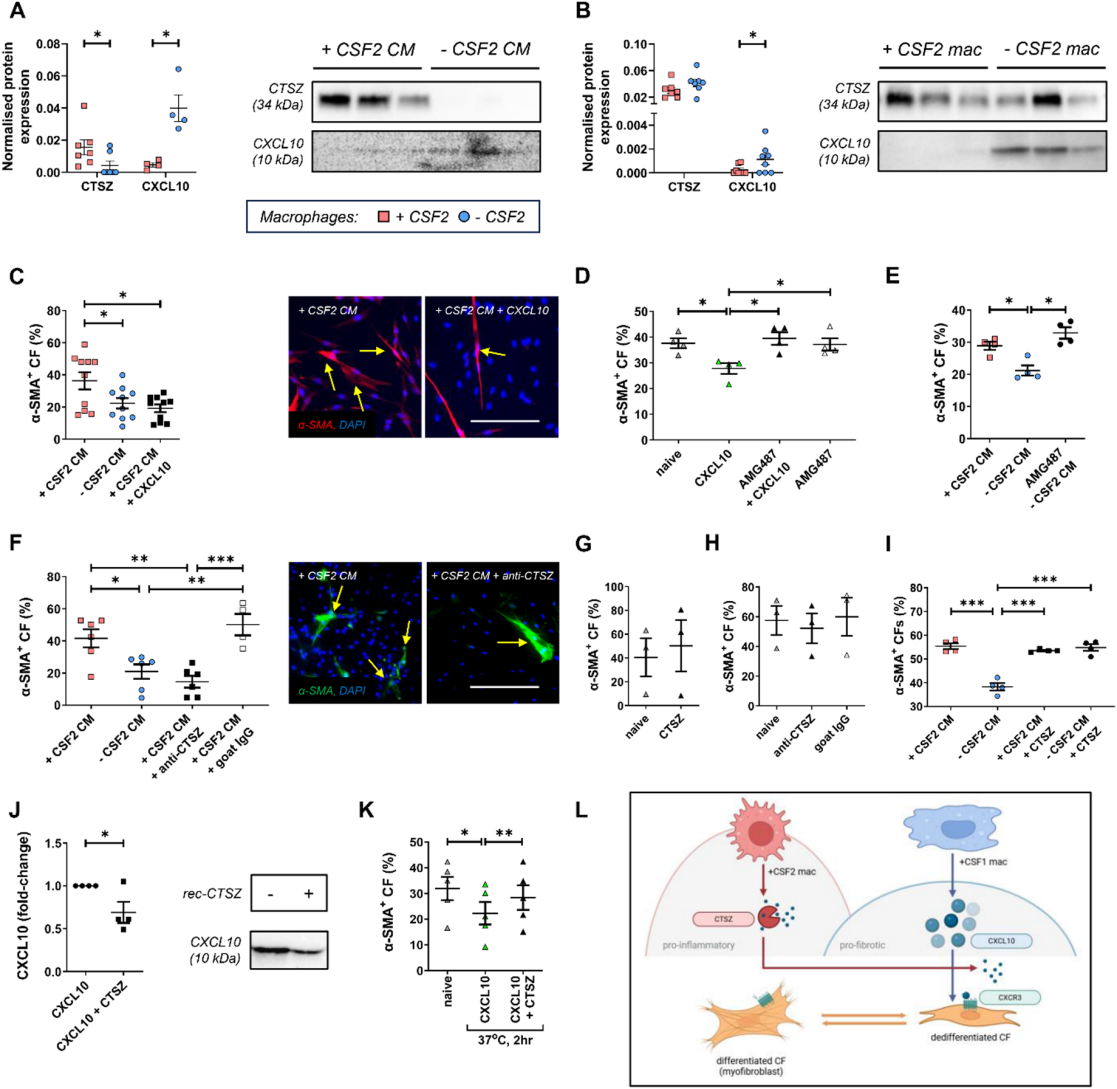
Loss of CSF2 macrophage polarisation promotes CXCL10 expression and CXCR3-induced cardiac fibroblast dedifferentiation, while CSF2-macrophage CTSZ degrades CXCL10. Validation of CTSZ and CXCL10 expression in **(A)** + CSF2 or -CSF2 macrophage secretome (*n* = 7 and 4), and **(B)** cell lysates (*n* = 7 and 8). Quantification and representative images of α-SMA assessed through immunocytochemistry (red in panel C, green in panel F): **(C)** supplementing CXCL10 protein (50 ng/mL) in the + CSF2 secretome (*n* = 10); **(D)** effect of CXCL10 protein (50 ng/mL) and AMG487 (1 µM), a CXCR3 inhibitor, on naïve cardiac fibroblasts (*n* = 4); **(E)** effect of CXCR3 inhibition, through AMG487 (1 µM) on -CSF2 secretome effect on cardiac fibroblast α-SMA expression (*n* = 4); **(F)** inhibition of CTSZ protein (4 µg/mL) in the + CSF2 secretome (*n* = 6, IgG *n* = 4); **(G)** effect of CTSZ protein (6 ng/mL) (*n* = 3) or **(H)** inhibition of CTSZ protein (4 µg/mL) on naïve cardiac fibroblasts (*n* = 3); **(I)** supplementing CTSZ protein (6 ng/mL) in macrophage secretome (*n* = 4). **(J)** CTSZ cleavage of CXCL10 protein (*n* = 4) and **(K)** effect on cardiac fibroblast α-SMA expression, assessed through immunocytochemistry (*n* = 5). **(L)** Proposed CTSZ/CXCL10-mediated mechanism in crosstalk between pro-inflammatory (CSF2) or pro-fibrotic (CSF1) macrophages and cardiac fibroblasts; generated using bioRender (https://www.biorender.com). Statistical significance is reported as *P < 0.05, **P < 0.01, or ***P < 0.001, using paired Students t-test to panels A, B, and J; ordinary one-way ANOVA, to panels C-F, and I, or repeated measures one-way ANOVA, to panel K, with Tukey’s multiple comparisons post-hoc. Scale bar represents 200 µM and is applicable to panels C and F.

As the secretome from macrophages with and without CSF2 signalling exhibited divergent effects on cardiac fibroblast differentiation towards a myofibroblast phenotype, we performed proteomics to elucidate the molecular mechanisms that facilitate macrophage crosstalk with cardiac fibroblasts. Comparative proteomics analysis between the + CSF2 and -CSF2 macrophage secretome revealed 64 differentially expressed proteins (*p* < 0.05 and LogFC > 1.5/<-1.5 cut-offs: Supplementary Figure S5A). Associated Reactome pathway analysis revealed multiple salient pathways which were significantly enriched and associated with downstream CSF2 signalling (JAK-STAT pathway) alongside collagen and ECM remodelling (Supplementary Figure S5B). Amongst the differentially expressed proteins (Supplementary Figure S5C) considered to harbour the potential to modulate cardiac fibroblast phenotype, Western blotting confirmed decreased Cathepsin Z (CTSZ) expression, alongside increased C-X-C Motif Chemokine Ligand 10 (CXCL10) levels in the secretome from -CSF2 macrophages compared to + CSF2 macrophage secretome (Fig. 5A) and elevated CXCL10 levels in cell lysates (Fig. 5B).

To explore a direct role for macrophage-derived CXCL10 in the intercellular communication and regulation of cardiac fibroblast function, the secretome from + CSF2 macrophages was supplemented with recombinant CXCL10 protein, which promoted changes associated with myofibroblast dedifferentiation, as observed through diminished α-SMA expression equivalent to that seen in -CSF2 macrophage secretome-treated fibroblasts, when compared to + CSF2 macrophage secretome alone (Fig. 5C). Furthermore, exogenous CXCL10 protein reduced α-SMA expression in cultured cardiac fibroblasts; this effect was blocked through pre-incubation with AMG487, an inhibitor for the CXCL10 cognate receptor CXCR3 (Fig. 5D). Moreover, AMG487 abrogated the suppressive action of -CSF2 macrophage secretome upon myofibroblast dedifferentiation (Fig. 5E), supporting a pivotal role of CXCL10/CXCR3 signalling in macrophage-myofibroblast intercellular communication to promote dedifferentiation and associated favourable scar remodelling and resolution. Recombinant CXCL10 protein had the same effects in alternative approaches when utilising macrophage-cardiac fibroblast co-cultures (Supplementary Figure S6A).

Similarly, inhibition of CTSZ (which was increased within the + CSF2 macrophage secretome) using a neutralising antibody decreased α-SMA expression in cardiac fibroblasts, compared to + CSF2 macrophage secretome alone (Fig. 5F). Equally, inhibiting CTSZ in macrophage-cardiac fibroblast co-cultures resulted in comparable effects (Supplementary Figure S6B). Given cardiac fibroblasts in culture predominantly acquire a myofibroblast phenotype, addition of recombinant CTSZ protein to naïve cells did not further influence α-SMA expression (Fig. 5G), and no difference was observed when neutralising CTSZ (Fig. 5H); together suggesting that CTSZ effects are indirect and mediated through interactions within the macrophage secretome. Moreover, recombinant CTSZ protein abrogated the effects of the -CSF2 macrophage secretome, with α-SMA expression comparable to the + CSF2 macrophage secretome (Fig. 5I).

Chemokines such as CXCL10 can be proteolytically processed by proteases, including matrix metalloproteinases (MMPs)^35,36^ and cathepsins^37^, resulting in activity modulation, inhibition or degradation^38–40^. Correspondingly, we explored the effect of CTSZ on CXCL10 stability, and we provide direct evidence for in vitro cleavage of CXCL10 (Fig. 5J), which results in abrogation of its effects on α-SMA expression (Fig. 5K), suggesting that the cleavage products of CXCL10 are inactive towards indicators of myofibroblast dedifferentiation. These findings suggest CSF2-mediated signalling in macrophages induces upregulation of CTSZ, which can subsequently abrogate the potentially beneficial effects of CSF1-produced CXCL10 on characteristics associated with myofibroblast dedifferentiation and reparative cardiac fibrosis and remodelling (Fig. 5L).

### CSF2RA Inhibition reduced cathepsin Z and concomitantly increased CXCL10 expression during reparative cardiac remodelling

Our proteomics and in vitro data revealed CSF2-mediated signalling can modulate macrophage secretion of anti-inflammatory and pro-reparative factors, highlighting a CTSZ/CXCL10-dependent mechanism, which governs crosstalk between macrophages and fibroblasts, with pertinent connotations for wound healing. Accordingly, to assess the in vivo relevance of the compromised reparative response seen in CSF2-polarised macrophages, we evaluated the expression of CTSZ and CXCL10 in macrophage-rich regions of hearts with cardiac injury from control and CSF2RA inhibitor-treated mice.

**Fig. 6:**
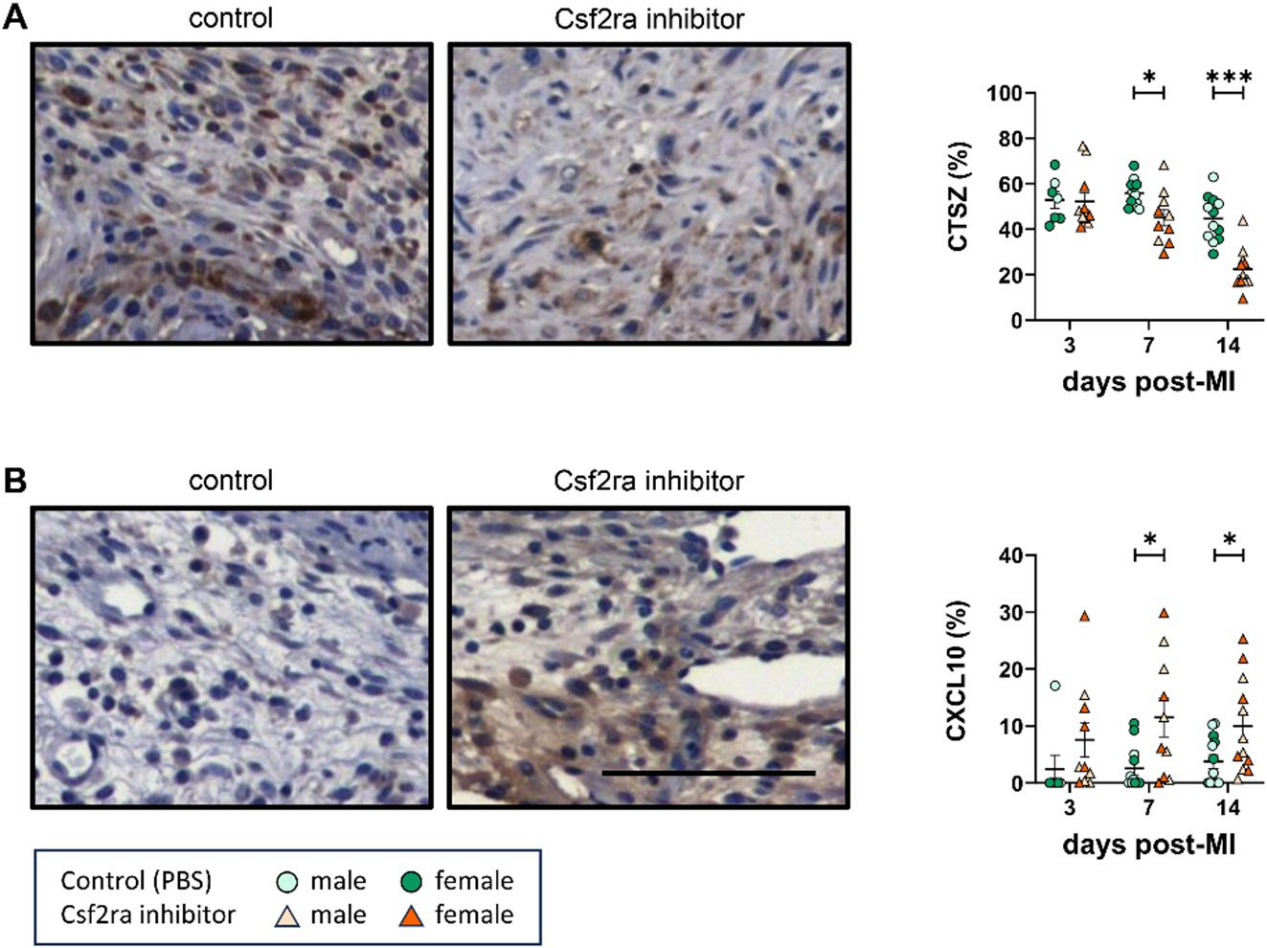
CSF2RA inhibition reduced Cathepsin Z and concomitantly increased CXCL10 expression within infarcts. Quantification and representative images of **(A)** CTSZ (*n* = 7–12) and **(B)** CXCL10 protein expression (*n* = 7–13) in CSF2RA inhibitor-treated and control mice, as assessed in correspondingly immunolabelled hearts. Statistical significance is reported as *P < 0.05 or ***P < 0.001, using unpaired Students t-test. Black scale bar represents 200 µM and is applicable to both panels.

Examination of serial myocardial cross-sections revealed macrophage expression of CTSZ and CXCL10 within remodelling regions at 7-days and 14-days post-cardiac injury (Supplementary Figure S7). Additionally, the regions of remodelling were enriched with a CXCR3-expressing population of cardiac fibroblasts, in comparison to healthy remote regions (Supplementary Figure S8). Consistent with our in vitro observations, we detected a significantly lower percentage of CTSZ-positive cells (Fig. 6A) alongside concomitantly increased number of CXCL10-positive cells (Fig. 6B) within areas of cardiac injury and remodelling in CSF2RA inhibitor-treated mice. Ancillary immunolabelling of CXCL10 with CD68-macrophages showed a significant increase in CXCL10-positive macrophages within the cardiac injury sites of CSF2RA inhibitor-treated mice (Supplementary Figure S9A); further strengthening the in vivo relevance of CSF2 signalling on macrophage phenotype and CXCL10 expression. No significant differences were observed in the presence of CTSZ-positive macrophages (Supplementary Figure S9B). Collectively, these proof-of-principle pre-clinical findings corroborate the in vivo studies with the in vitro proposed mechanism, suggesting a critical role for CSF2/CSF2RA-mediated macrophage polarisation in the communication between macrophages and fibroblasts in regulating pathological cardiac remodelling following acute myocardial injury.

## Discussion

CSFs are proposed to direct macrophage polarisation towards pro-inflammatory (CSF2-driven) and pro-fibrotic (CSF1-driven) phenotypes^10^. Relatedly, during the biphasic post-MI immune response^6^, pro-inflammatory and anti-inflammatory macrophages are considered to differentially regulate the transition of cardiac fibroblasts towards a myofibroblast phenotype and subsequent adverse post-MI cardiac fibrosis and remodelling. Moreover, abruptly after MI and associated ischaemia, cardiac fibroblasts release CSF2 (GM-CSF) to orchestrate the recruitment of inflammatory cells (monocytes and neutrophils) to the infarcted myocardium with detrimental effects on scar formation resulting in increased susceptibility to cardiac rupture^13^. Furthermore, hypercholesterolemic mice (such as Apoe-deficient) display monocytosis through heightened CSF2 responsiveness^13,41^ and exhibit impaired infarct healing due to dysregulated resolution of inflammation^42^. Given the proposed role for CSF2 in generating pro-inflammatory macrophages alongside post-MI inflammatory responses and cardiac repair, identifying key signalling mechanisms and related factors that affect myocardial healing are of clear therapeutic importance.

However, previous studies have been unable to delineate CSF2-specific effects from those of IL-3 and IL-5 given that Csf2rb-deletion methods were utilised^13^. Accordingly, our study, for the first time, evaluated the effect of direct pharmacological inhibition of the CSF2-specific receptor subunit, CSF2RA, on pathological cardiac remodelling in a mouse model of cardiac dysfunction and fibrosis. Ancillary in vitro approaches and proteomic analysis were deployed to provide mechanistic insight. CSF2RA loss-of-function through pharmacological inhibition, promoted a pro-fibrotic reparative macrophage phenotype and accelerated the dedifferentiation of cardiac myofibroblasts and the angiogenic response. Concurrently, CSF2RA loss-of-function accelerated maturation remodelling, and resolution of the injury site and consequently limited the scar expansion to the border zone, resulting in improved cardiac morphology and contractility. Mechanistically, we found that in the absence of CSF2-mediated signalling, macrophages impart divergent effects upon characteristics associated with cardiac myofibroblast dedifferentiation in the context of post-cardiac injury remodelling and resolution. We elucidated that CSF2 receptor-induced signalling in macrophages exerts detrimental effects upon pathological cardiac remodelling, through hindering myofibroblast dedifferentiation during the resolution period, through a CTSZ/CXCL10-mediated mechanism. Indeed, our findings reveal the CSF2-dependent CTSZ/CXCL10-mediated mechanism sustained the myofibroblast phenotype with migratory and contractile capacity. Indeed, corroborating evidence demonstrated that cardiac fibroblast contraction contributes to increased mechanical stress of the ECM leading to perpetual myofibroblast differentiation^43^. Furthermore, increased myofibroblast migration and concomitant expansion of the collagenous tissue to remote healthy regions of the myocardium, increases myocardial scar stiffness with detrimental implications to heart function^30,44^.

During the post-MI inflammatory response, macrophages become polarised according to their exposure to intricate cues in the microenvironment. The macrophage transcriptome undergoes significant changes, and polarised macrophages sequentially adopt distinct phenotypes^45^, with subsequent changes translating to their proteome and secretome. Although transcriptional changes between CSF-differentiated macrophage subsets have been described^11,12^, here we characterised the secretome of macrophages with and without CSF2-mediated polarisation. The secretome of CSF2-polarised macrophages was significantly enriched for proteins associated with ECM remodelling, including several MMPs and cathepsins. While a role for MMPs in ECM turnover, cardiac fibrosis, and remodelling, secondary to MI have been described^46^, cathepsins are less studied. During post-MI remodelling, particularly the pro-inflammatory phase, increased cathepsin expression and activity has been detected, deriving from monocyte/macrophages^6,47^. Although commonly found within specialised, highly acidic, lysosomal compartments^48^, cathepsins are also found in the extracellular environment, supporting our macrophage secretome findings. Despite their susceptibility to alkaline conditions, cathepsins remain active within the extracellular space^49,50^, especially the infarcted area of the myocardium with a high density of dying cardiomyocytes and reactive oxygen species, fostering an acidic microenvironment and maintaining cathepsin activity^51^. Extracellular localised cathepsins can cleave cell adhesion molecules and ECM components to modulate cell motility^52^, and also process chemokines and therefore regulate their activity^37^. Accordingly, our reported elevation of CTSZ within the secretome of CSF2-polarised macrophages and subsequent effects on fibroblast behaviour, support a potential role in pathological cardiac remodelling. Relatedly, CTSZ levels were increased within the blood of patients with an ST segment elevation MI (STEMI)^53^ or a NSTEMI^54^, with CTSZ expression positively correlated with adverse outcomes. Our study identified that CTSZ cleaves CXCL10 and abrogates its actions upon cardiac fibroblasts, with potentially detrimental effects for reparative cardiac remodelling.

Contrastingly, CXCL10 was abundantly expressed and released/secreted from macrophages exposed to CSF1 alone, in comparison to CSF2-mediated polarisation. CXCL10 is not homeostatic; instead it is a chemokine inducible upon inflammation, with increased circulating levels reported in multiple cardiovascular diseases, including atherosclerosis and MI^55^. Indicative of an underlying pathophysiological relationship between CXCL10 levels and cardiac remodelling post-MI, an acute increase in CXCL10 expression was reported in a canine post-MI-reperfusion model^56^, and in patients with heart failure compared to healthy subjects^57^. Additionally, ischaemia/reperfusion experiments in CXCL10-deficient and wild-type mice suggested that CXCL10 promotes a favourable post-MI reparative response, partly through enhancing myofibroblast dedifferentiation as observed through their reduced α-SMA positivity and migratory capacity, and their reduced wound-contracting properties^58^. Building on previous knowledge, our findings suggest a co-ordinated response which ensures CSF1-directed macrophages can promote CXCL10/CXCR3-mediated myofibroblast dedifferentiation adjacent to areas of cardiac injury (such as an infarct) to promote reparative healing, a process which is facilitated by low CTSZ levels, contrary to the situation when CSF2-mediated macrophage signalling persists.

We demonstrate that CSF2RA loss-of-function promoted a reparative pro-remodelling macrophage phenotype in vivo, associated with increased CD206 and CXCL10 expression, and reduced iNOS and CTSZ levels. From a biomarker perspective, we evaluated the effect of CSF2RA inhibition upon plasma chemokines and cytokine levels. In line with CSF2RA inhibition fostering a reparative macrophage phenotype during the remodelling phase post-cardiac injury, several anti-inflammatory and reparative mediators were upregulated in the plasma of CSF2RA inhibitor-treated mice, including IL-10, CCL1, CCL4, and CCL12. IL-10 is the prototypical cytokine for anti-inflammatory (M2) macrophage polarisation, and previous studies have shown that exogenous IL-10 stimulated anti-inflammatory macrophage polarisation and resulted in improved survival and cardiac function in mice post-MI^24^. CCL1 is a pleiotropic chemokine and expedites anti-inflammatory macrophage polarisation, especially generation of M2b macrophages, which also display heightened CCL1 production^59^. Furthermore, CCL1 and CCL4 promote adaptive immune responses and facilitate the recruitment of regulatory T-cells^60–62^, which are involved in ischaemic heart disease and are associated with improved cardiac function and reduced fibrosis post-MI in mice^60,63^. Finally, CCL12 (while having some opposing effects in periodontitis-complicated MI^64^) is associated with pro-fibrotic responses on fibroblasts and progenitor cells in the heart and lung^65–67^. Unexpectedly, CSF2RA inhibition also raised levels of some pro-inflammatory mediators, including CCL2 and IL1β, although this observation may represent a compensatory response or may be due to the accumulation of cardiac fibroblasts^13^. Overall, these findings suggest that the favourable effects of CSF2RA inhibition observed upon cardiac injury remodelling could be detected systemically, through elevated levels of circulating chemokines and cytokines that are associated with enhanced reparative fibrosis and improved post-MI recovery.

Interestingly, the observed effects in FS (Fig. 2B), replacement fibrosis (Fig. 2D), and macrophage accumulation (Fig. 3B) were more prominent in female mice, even though male mice were also benefited. In humans, the location of *CSF2RA* within the pseudo-autosomal region (PAR) of the X chromosome^68,69^ suggests that CSF2RA is potentially expressed two-fold greater in females than males. Moreover, the homologous *Csf2ra* gene in mice is expressed in the autosomal X chromosome region, rather than pseudo-autosomal region, suggesting that female mice will have twice as much Csf2ra, compared to male mice^70^; potentially explaining the lesser response in male mice. Nonetheless, male mice exhibited similar effects upon scar cellular (cardiac fibroblasts, macrophages, and capillaries) and extracellular (collagen maturation) composition, further strengthening the translational potential of CSF2RA inhibition in both sexes. Additionally, part of the sex-biased effects may be dependent on CXCL10/CXCR3 signalling, as the *CXCR3* gene is located within the X chromosome and is overexpressed in women^71,72^. Relatedly, in women, higher serum CXCL10 levels corelated with protection following a first-ever MI and better prognosis, in comparison to men^73^. However, infarcted regions from female and male mice subjected to CSF2RA inhibition showed increased cardiac fibroblast infiltration and density, which is associated with promotion of wound healing and a reduction in cardiac rupture post-MI in mice^74^. Similarly, while macrophage infiltration and density were increased, with accentuated macrophage influx correlated with cardiac rupture^74^, CSF2RA inhibition permits the macrophages accumulating in the infarct to acquire an pro-fibrotic phenotype, as indicated by the reduced CTSZ and increased CXCL10 levels, enabling their pro-fibrotic actions^12^.

Since CSF2 has an attributed role in emergency haematopoiesis and ensuing increased production of innate immune cells during infection, CSF2 therapy was considered a potential beneficial approach to protect the myocardium from injury after infarction and subsequent reperfusion but yielded predominantly ambivalent or deleterious results^75^. Pre-clinical post-MI studies have shown no effect of subcutaneous CSF2 administration upon infarct size or cardiac function in pigs^76^, while systemic (intraperitoneal) delivery of CSF2 induced infarct expansion in association with a pro-inflammatory and proteolytic milieu alongside a delayed fibrotic response, during the early phase of MI in rats^77,78^. Supporting, plasma CSF2 levels are markedly higher in acute MI patients with subsequent heart failure complications, compared to patients with uncomplicated acute MI or healthy subjects^14^. Clinical studies in humans were driven by the hypothesis that combined intra-coronary injection and systemic delivery of CSF2 would promote arteriogenesis to facilitate collateral flow in patients with obstructive coronary artery disease^79,80^. However, a clinical trial evaluating systemic application of CSF2 in patients with coronary artery disease was halted as 2/7 patients, but none within the placebo group, experienced a recurrent acute coronary event, attributed to atherosclerotic plaque rupture^81^. Consistent with a detrimental role for CSF2 signalling during post-MI responses, our in vivo approach demonstrated CSF2RA governs the pernicious function of macrophages after infarction, and CSF2RA inhibition accelerated reparative cardiac remodelling in mice, through favourable effects on scar cellular and collagen composition during the early phase of acute myocardial injury, resulting in improved cardiac function. Although previous evidence in mice has demonstrated modulation of CSF2 levels (Csf2-deletion) or receptor-mediated signalling (Cs2rb-deletion) imparts beneficial effects upon cardiac repair after infarction^13^, it is evident that effects on other CSF2RB signalling pathways cannot be distinguished. This is due to CSF2RB being also a co-receptor for IL-3 and IL-5^12^. Our experimental design benefits from utilising CSF2RA as a target due to its specificity for CSF2.

A limitation of our study is the use of a refined LAD ligation-induced injury to comply with the Animals in Science Regulation Unit (ASRU) guidance and limit the occurrence of sudden death. Accordingly, the refined model does not produce an infarct as large as commonly described in LAD ligation models^82^. Nonetheless, when compared to sham-operated animals, we detected marked cardiac fibrosis, myofibroblast activation, and macrophage accumulation (Supplementary Figure S10A-D) alongside a deterioration in cardiac function (Supplementary Figure S10E-F) 7- and 14-days post-myocardial injury through LAD ligation. In alignment with these data, CSF2RA inhibition did not affect the cardiac function of the healthy (sham) hearts, which displayed preserved contractile capacity and minimal cardiac fibrosis at days 14 and 28 post-surgery (Supplementary Figure S11). Our refinement provides a valuable alternative model of cardiac dysfunction and fibrosis, without the marked death rates associated with full-wall MI models. In support of the rigor of the deployed model, other groups have reported the induction of reproducible, small, transmural infarcts, by ligating a more distal portion of the LAD^83^. We have demonstrated that absence of CSF2-mediated signalling enhanced cardiac function and improved post-MI wound healing in mice subjected to permanent ligation of the distal LAD. Extrapolating these results to a range of infarct sizes is important to ensure optimal wound healing and further strengthen the translatability of our findings. In support, previous evidence shows that in the commonly used model of severe ischaemia through permanent LAD ligation, Csf2^−/−^ mice presented with reduced incidence of cardiac rupture – suggesting adequate replacement fibrosis – and improved mortality^13^. The pharmacological CSF2RA inhibition provides improved therapeutic potential. While beyond the scope of the current study, it is possible very early effects upon neutrophil infiltration immediately after myocardial injury maybe afforded through CSF2RA inhibition, alongside associated abrogation of neutrophil-related proteolysis. It has been demonstrated that neutrophils orchestrate acute post-MI inflammatory responses, but their numbers are markedly diminished after the first 24 hours^18,19^. Accordingly, from an intervention and therapeutic perspective, we focused upon the role of macrophages, which are the main inflammatory cell type that coordinates the long-term wound healing process 24-hours post-MI. Lastly, while the data suggests the beneficial effects afforded through CSF2RA inhibition were predominantly upon recruited monocyte-derived macrophages, we can discount potential effects upon resident cardiac macrophages.

In summary, we demonstrate that CSF2RA-mediated macrophage polarisation generates a distinct subset of macrophages which drives pro-inflammatory responses post-cardiac injury, through a CTSZ/CXCL10-mediated mechanism to modulate cardiac fibroblast behaviour and thereby preventing the resolution of pathological cardiac remodelling. The pre-clinical intervention through CSF2RA inhibition revealed accelerated reparative remodelling, due to enabling favourable CSF1-driven pro-fibrotic responses and thereby highlights a direct translational potential for promoting post-MI recovery and limiting subsequent heart failure risk.

## Methods

### Study approval

The housing and care of the animals and all the procedures used in these studies were performed in accordance with the ethical guidelines and regulations of the University of Bristol and the UK Home Office (PPL No. PA91C29DB). All the in vivo study protocols were reviewed and approved by the Animal Welfare and Ethics Review Body (AWERB), University of Bristol. Adherence to the ARRIVE guidelines (https://arriveguidelines.org) for the reporting of animal in vivo experiments was also followed. The investigation also conforms to the guidelines from Directive 2010/63/EU of the European Parliament on the protection of animals used for scientific purposes. Studies utilising human cells and blood were reviewed and approved by the Research Governance and Ethics Committee of the University of Bristol and NHS England. Human participants signed an informed consent form, under Research Ethics Committee reference 10/H0107/32, prior to donating blood. The studies were conducted in accordance with the Declaration of Helsinki principles.

### In vivo mouse study

*CSF2RA inhibition*. C57Bl/6NCrl mice (sourced from Charles River) were administered intraperitoneally a CSF2RA peptide inhibitor, (E21R at 1 µg/mL, cat. no. 13702-01, Bio-Synthesis) or PBS (controls), immediately after LAD ligation, and subsequently on days-3, -7, -14, and − 21 after LAD ligation. *Cardiac injury model in mice*. Mice were subjected to MI via permanent LAD ligation. To comply with Animals in Science Regulation Unit (ASRU) guidance, where death is not permitted as an end-point, we used a refined method where ligation was undertaken within a more distal portion of the LAD. While this results in smaller infarct sizes (in comparison to previous studies^84^, a marked improvement in survival rates (96%) is observed compared to the standard approach (60%) in C57Bl/6 mice^82^. Briefly, mice were anaesthetised with isoflurane and oxygen (2.5% and 1 L/min) with subsequent intubation and mechanically ventilated with a pressure-regulated small animal ventilator MiniVent Model 845 (catalogue number 73 − 0044, Hugo Satchs Electronik). Mice underwent thoracotomy and the pericardium was incised to expose the heart; the LAD coronary artery was tied using an 8 − 0 Ethilon suture, and the intercostal space and skin were sutured, before mice were allowed to recover. Echocardiographic analysis was performed using a Vevo 3100 (Fujifilm VisualSonics) and analysed with Vevo LAB 5.7.1 (Fujifilm VisualSonics). Postoperative analgesia was performed through intraperitoneal injection of buprenorphine (Temgesic; 0.1 mg/kg), immediately after surgery and animals closely monitored for 24 h and subsequent pain relief delivered if necessary. At the relevant time-point, mice were anaesthetised by intraperitoneal injection of sodium pentobarbitone (500 mg/kg of bodyweight) before exsanguination by perfusion via the abdominal aorta with PBS at a constant pressure of 100 mmHg, with outflow through the incised jugular veins. Prior to exsanguination, a blood sample was drawn via the abdominal aorta. This was followed by constant pressure perfusion with 10% formalin, after which hearts were collected for histological analysis. Hearts were processed for histology via dehydration, clearing, and infiltration with paraffin, and 3 µM thick sections were cut for downstream analyses. The researchers remained blinded to the genotypes until analysis was complete. Both male and female mice were used in all experiments, and mice were randomly assigned to the groups using the NC3Rs Experimental Design Assistant Tool.

### Bio-Plex multiplex immunoassays

Plasma was isolated following centrifugation for 5 min at 4 °C (12,000 rpm). Mouse plasma chemokine and cytokine levels were quantitatively assessed using a Bio-Plex Pro Mouse Multi-Plex Assay^85^, in accordance with the manufacturer’s protocol (Bio-Rad). Briefly, 50 µL of diluted plasma samples (1:5) were incubated with 50 µL of fluorescently dyed magnetic beads for 30 min at room temperature on a plate shaker (850 rpm). After 3 washes with Bio-Plex wash buffer, the beads were incubated with 25 µL of biotinylated detection antibodies for 30 min at room temperature on a plate shaker (850 rpm). After 3 washes with Bio-Plex wash buffer, the beads were incubated with 50 µL of diluted streptavidin-phycoerythrin (SA-PE) conjugate for 10 min at room temperature on a plate shaker (850 rpm). After 3 washes with Bio-Plex wash buffer, the beads were resuspended in Bio-Plex assay buffer, and the plate was read on a Luminex 200 (Bio-Rad). Data were analysed using the Bio-Plex Manager 6.1.

### Histochemistry

To assess myocardial replacement (infarct) and interstitial (border zone) fibrosis, trichrome staining was performed using a Masson’s trichrome staining kit (catalogue number HT15, Sigma-Aldrich), according to the manufacturer’s instructions. For each mouse heart, total area of replacement fibrosis and percentage of interstitial fibrosis were quantified within two sections, distanced 50 µM apart, and the average value is presented. To assess nucleated cell density, haematoxylin and eosin (H&E) staining was performed using a Shandon Varistain 24 − 4 Automatic Slide Stainer (Thermo Scientific, 74200103). Fibrillar collagen fibre content was assessed using a Picrosirius Red stain kit for cardiac muscle (catalogue number, ab245887, Abcam), according to the manufacturer’s instructions; images were analysed under linearly polarised light to assess thickness and maturation of fibrillar collagen^86^. The total area of red (old) and green (new) collagen fibres was quantified using Fiji ImageJ 1.54 and was expressed as a percentage of the total area of collagen fibres.

### Immunohistochemistry

The expression and localisation of proteins of interest were investigated with indirect immunohistochemistry and brightfield or fluorescent visualisation. Briefly, slides were dewaxed in Clearene and rehydrated in graduated alcohol, endogenous peroxidase activity was blocked using 3% (v/v) H_2_O_2_ (catalogue number H/1750/17, Fisher Chemical), and heat-mediated antigen retrieval was performed in 10 mM citrate buffer, pH 6.0. Sections were blocked with 20% (v/v) appropriate animal serum or Trident Universal Protein Blocking Reagent (animal serum free) (catalogue number GTX30963, GeneTex) and subsequently incubated with the appropriate primary antibody (Supplementary Table S4). Concentration- and species-matched non-immune IgG isotype controls were used as a negative control (Supplementary Figure S12). Brightfield visualisation was performed with biotinylated secondary antibodies, ExtrAvidin-peroxidase (catalogue number E2886, Sigma-Aldrich), and the peroxidase substrate, SigmaFast 3,3’-diaminobenzidine (DAB) (catalogue number D4293, Sigma-Aldrich), and counter-stained with the nuclear stain Gill’s No. 2 haematoxylin (catalogue number GHS2128, Sigma-Aldrich). Fluorescent visualisation was performed with fluorescently-conjugated secondary antibodies and mounted with ProLong Gold Antifade Mountant with DAPI (catalogue number P36931, Invitrogen). Immunolabelling of the mouse hearts with antibodies raised in mice was performed using the fluorescein mouse-on-mouse (M.O.M.) immunodetection kit (catalogue number FMK-2201, Vector Laboratories), according to the manufacturer’s instructions. Cellular composition measurements were performed across three sections for each mouse infarct, and the average values are provided for each mouse. Presented results are normalised to the total number of cells (nuclei per field-of-view), unless otherwise specified for results normalised to the total number of fibroblasts (vimentin), macrophages (CD68), or endothelial cells (IB4).

### Cell culture

*Human primary peripheral blood mononuclear cells* were isolated from consenting healthy male and female participants, using SepMate-50 tubes (catalogue number 85450, STEMCELL Technologies) filled with Ficoll Paque PLUS (catalogue number 17144002, Cytiva), washed in Hanks’ Balanced Salt Solution (HBSS) without calcium/magnesium (catalogue number 14170088, Gibco) and cultured in RPMI-1640 medium with 10% (v/v) FBS for 2 h, to isolate monocytes by adhesion. Monocytes were cultured for 7 days with the appropriate CSFs: 40 ng/mL CSF1 (M-CSF) (catalogue number 130-096-491, Miltenyi Biotec) or 20 ng/mL CSF1 (M-CSF) and 20 ng/mL CSF2 (GM-CSF) (catalogue number 130-095-372, Miltenyi Biotec), to differentiate into CSF1 (M-) or CSF2 (GM-) macrophage subsets, respectively. *Primary human cardiac fibroblasts*, isolated from the ventricles of the adult heart, were commercially bought (catalogue number C-12375, PromoCell), and cultured in Fibroblast growth medium 3 (catalogue number C-23025, PromoCell) supplemented with Supplement Mix (catalogue number C-39345, PromoCell), as per manufacturer’s instructions. Cardiac fibroblasts (10 × 10^4^ cells/mL) were quiesced for 24 h in serum-free DMEM and subsequently cultured in 24-well plates with macrophage conditioned medium or co-cultured with an equal number of CSF1 (M-) or CSF2 (GM-) macrophages.

### Immunocytochemistry

The presence and localisation of specific markers were assessed with immunocytochemistry. Briefly, cells were fixed with 3% (w/v) PFA/PBS, permeabilised with 0.2% (v/v) Triton X-100 in PBS (catalogue number X100, Sigma-Aldrich), blocked with 20% (v/v) animal serum in PBS (catalogue number S26, Sigma-Aldrich) and probed with appropriate primary antibodies (Supplementary Table S5) and fluorescently-conjugated secondary antibodies; concentration- and species-matched non-immune IgG isotype controls were used as a negative control (Supplementary Figure S12). Cells were mounted with ProLong Gold Antifade Mountant with DAPI (catalogue number P36931, Invitrogen) and imaged.

### Western blotting

Protein expression was evaluated with Western blotting in cell-conditioned medium, concentrated with centrifugation using Amicon Ultra-0.5 centrifugal filter 10 kDa devices (catalogue number UFC501096, Merck Millipore), or cell lysates, prepared in 50 mM Tris-HCl (pH 6.8), 1% (w/v) SDS, and 10% (v/v) glycerol. Briefly, samples were heat-denaturised with Laemmli sample buffer (catalogue number S3401, Sigma-Aldrich), and an equal amount of lysate protein or equal volume of concentrated conditioned medium was loaded per lane on Mini-PROTEAN TGX Stain-Free Precast Gels (catalogue number 4568086, Bio-Rad). Gels were subjected to electrophoresis and total protein was visualised using the stain-free gel technology (Bio-Rad), prior to protein transfer on 0.2 µM nitrocellulose membranes (catalogue numbers 1704159, Bio-Rad). Nitrocellulose membranes were blocked with 5% (w/v) non-fat dried milk in tris-buffered saline supplemented with 0.1% (v/v) Tween-20 (TBS-T, 20 mM Tris, 137 mM NaCl, pH 7.6) and subsequently probed with the appropriate primary antibodies (Supplementary Table S6) and HRP-conjugated secondary antibodies. Enhanced chemiluminescent HRP-substrate (catalogue number WBLUF0500, Merck Millipore) was used and protein detection was performed with a Chemidoc MP Imaging System (Bio-Rad). Relative densitometry was performed with Image Lab Software, version 6.0.1 (Bio-Rad). The original full-length Western blots are shown in Supplementary Figures S13-20.

### Quantitative real-time polymerase chain reaction

Expression of mRNA was investigated with quantitative real-time polymerase chain reaction (qPCR). Cells were lysed in QIAzol lysis reagent (catalogue number 79306, Qiagen) and collected in RNAse-free Eppendorf tubes. Total mRNA content was purified using a miRNeasy mini kit (catalogue number 217004, Qiagen) and reverse transcription was performed using a high-capacity RNA-to-cDNA kit (catalogue number 4387406, Applied Biosystems), according to the manufacturer’s instructions. qPCR was performed using the LightCycler 480 SYBR Green I Master ready-to-use hot start reaction mix (catalogue number 04707516001, Roche) for SYBR Green I-based real-time PCR using the LightCycler 480 Instrument (Roche) and results are presented as copy numbers for each transcript (Supplementary Table S7).

### Fibroblast contraction

Contraction of cardiac fibroblasts was assessed using a Cell Contraction Assay kit (catalogue number CBA-201, Cell Biolabs). The assay is based on a two-step model of collagen contraction following mechanical stress development^87^. Briefly, pooled cardiac fibroblasts (2 × 10^5^ cells) were quiesced and pre-treated with macrophage-conditioned medium for 24 h. Subsequently, a cardiac fibroblast-collagen lattice was prepared, as per manufacturer’s instructions, and cultured for 48 h, allowing mechanical stress to develop, before release from the wells to initiate contraction. Gels were imaged at 24 h and the contraction (% of original gel area) was calculated. Additionally, cardiac-fibroblast-populated free-floating gels were used to assess activation of human cardiac fibroblasts in a 3D, collagen-based environment, in comparison to plastic/glass surfaces that result in fibroblast activation in vitro^30,34^. Briefly, quiesced cardiac fibroblasts (10 × 10^5^ cells/mL) were resuspended in Temin’s MEM (2X) (catalogue number 21935028, Gibco) and mixed with collagen type 1 (catalogue number A1048301, Gibco), final concentration 2 mg/mL. Following polymerisation in a 24-well plate, gels were released and transferred into a 12-well plate. The free-floating collagen pads were used for assessment of cell contraction (48 h), confocal microscopy of collagen pads following α-SMA immunolabelling, and extraction of cardiac fibroblasts, following type I collagenase dissociation and centrifuged on a slide using a Shandon Cytospin 4 (Thermo Scientific); cell smears were immunolabelled for α-SMA.

### Fibroblast migration

Migration of cardiac fibroblasts was assessed using a scratch-wound assay of a monolayer of cells and measuring their capacity to repopulate the wound^88,89^. Pooled cardiac fibroblasts (5 × 10^4^ cells/well) were quiesced and subsequently subjected to wounding by performing two parallel scratches, gently washed in PBS and treated with macrophage-conditioned medium, supplemented with hydroxyurea (2 mM) to prevent cell proliferation; scratches were imaged for 48 h and cell migration (% coverage of initial scratch width) was calculated.

### In vitro invasion assay

Invasion of human monocyte-derived macrophages was assessed through invasion within Matrigel-coated transwells, as previously described^26^. Briefly, transwell inserts (8 µM pores) were coated with 25 µL of BD Matrigel matrix (BD Biosciences, Oxford). Macrophages (10^5^ cells/transwell in RPMI) were added on the Matrigel and treated with or without the CSF2RA inhibitor. RPMI was supplemented with 30ng/mL of recombinant monocyte chemoattractant protein-1 (MCP-1) and 30ng/mL of recombinant fractalkine (CX3CL1) (R&D Systems) was placed in the lower wells to induce transmigration/invasion. After 48 h, the cells on both the upper and lower surface of the membrane were fixed with 3% (w/v) PFA/PBS and stained with haematoxylin. The number of migrated/invaded cells expressed as a percentage of total cells.

### Cathepsin Z cleavage assay

Recombinant human CXCL10, 100 ng, (catalogue number 300-12-5, PeproTech) was incubated with or without recombinant human CTSZ, 10 ng, (catalogue number 934-CY-010, R&D) in distilled water (2 h, 37 °C). For Western blotting, following incubation, the reaction was stopped through the addition of an equal volume of Laemmli sample buffer (catalogue number S3401, Sigma-Aldrich) and heating (95 °C, 5 min). The samples were subjected to electrophoresis, as previously described, and the relative abundance of CXCL10 was estimated. For cell culture functional experiments, the reaction was kept under sterile conditions; following incubation, the reaction was used to treat human cardiac fibroblasts, as previously described^35–37,39,40^.

### Proteomics

Macrophage-conditioned medium (*n* = 2 male, *n* = 2 female) was concentrated with centrifugation using Amicon Ultra-0.5 centrifugal filter 10 kDa devices (catalogue number UFC501096, Merck Millipore) and incubated at 60 °C for 1 h with an equal volume of 100% 2,2,2- trifluoroethanol (TFE; catalogue number 96924-50ML-F, Sigma-Aldrich). Tandem Mass Tagging (TMT) quantitative proteomics were performed at the University of Bristol Proteomics Facility. Proteomic data were interrogated with the use of Ingenuity Pathway Analysis software (IPA; https://digitalinsights.qiagen.com/IPA), version 8.0 (Qiagen Bioinformatics).

### Statistical analysis

All values are presented as mean ± standard error of the mean (SEM). When comparing group means, groups were tested for normal distribution with D’Agostino-Pearson test. For normally distributed groups, a t-test, or ordinary one-way ANOVA with Tukey’s multiple comparisons post-hoc test was used. In vitro experiments performed with CSF2- or CSF1-macrophages and the corresponding secretomes were analysed with paired analyses. Statistical significance is reported as *P < 0.05, **P < 0.01, and ***P < 0.001. Statistical analyses were performed, and graphs were generated using GraphPad Prism 9.

## Supplementary Information

Below is the link to the electronic supplementary material.

Supplementary Material 1

## Data Availability

The datasets used and/or analysed during the current study are available from the corresponding author on reasonable request.
