# Supplementary material for: Suppression of CSF2RA macrophage polarisation impacts pathological cardiac remodelling in mice
Source: Sci Rep. 2026 Jan 9;16:3870. doi: 10.1038/s41598-025-33936-1 (PMC12852799; doi:10.1038/s41598-025-33936-1)
Supplement: Supplementary file 1 — Supplementary Material 1 [file 41598_2025_33936_MOESM1_ESM.docx]

Suppression of CSF2RA macrophage polarisation impacts pathological cardiac remodelling in mice

Georgios Kremastiotis^1^‡, Yong Li^2^, Andrew Bond^1^, Daire Shanahan^1^, Karina Di Gregoli^1^, Alastair W. Poole^2^, Sarah J. George^1^, Jason L. Johnson^1^*

^1^Laboratory of Cardiovascular Pathology, Bristol Medical School, Faculty of Health Sciences, University of Bristol, Bristol, UK. ^2^School of Physiology, Pharmacology & Neuroscience, Faculty of Life Sciences, University of Bristol, Bristol, UK

*Correspondence: Professor Jason L. Johnson ([jason.l.johnson@bristol.ac.uk](mailto:jason.l.johnson@bristol.ac.uk)), Laboratory of Cardiovascular Pathology, Translational Health Sciences, Bristol Medical School, Faculty of Health Sciences, University of Bristol, Level 7, Bristol Royal Infirmary, Bristol, BS2 8HW, UK; Tel. +441173423583.

‡Present address: National Heart and Lung Institute, Imperial College London, Sir Michael Uren Building, 86 Wood Lane, White City Campus, London W12 0BZ, UK

Running Title: CSF2RA signalling affects cardiac injury

**Supplementary Table S1.** Echocardiographic assessment of cardiac function of control and CSF2RA inhibitor-treated mice at 14 days post-MI. IVS, interventricular septum; LVID, left ventricular internal diameter; LVPW, left ventricular posterior wall thickness; EF, ejection fraction; FS, fractional shortening; LV Vol, left ventricular volume; d, diastole; s, systole.

| **day 14 post-MI** | **control** | **Csf2ra inh** | **p-value** |
| --- | --- | --- | --- |
| ***IVS;d (mm)*** | 0.93 ± 0.05 | 0.92 ± 0.06 | 0.9051 |
| ***IVS;s (mm)*** | 1.24 ± 0.04 | 1.32 ± 0.05 | 0.2115 |
| ***LVID;d (mm)*** | 3.84 ± 0.08 | 3.79 ± 0.10 | 0.7305 |
| ***LVID;s (mm)*** | 2.87 ± 0.10 | 2.64 ± 0.11 | 0.1488 |
| ***LVPW;d (mm)*** | 0.83 ± 0.04 | 1.024 ± 0.11 | 0.0868 |
| ***LVPW;s (mm)*** | 1.17 ± 0.05 | 1.42 ± 0.11 | *0.0429 |
| ***EF (%)*** | 50.45 ± 2.44 | 58.35 ± 2.67 | *0.0480 |
| ***FS (%)*** | 25.43 ± 1.45 | 30.54 ± 1.67 | *0.0366 |
| ***LV Vol;d (μL)*** | 63.92 ± 2.97 | 62.38 ± 3.98 | 0.7658 |
| ***LV Vol;s (μL)*** | 32.13 ± 2.51 | 26.42 ± 2.73 | 0.1545 |

**Supplementary Table S2.** Echocardiographic assessment of cardiac function of control and CSF2RA inhibitor-treated mice at 28-days post-MI. IVS, interventricular septum; LVID, left ventricular internal diameter; LVPW, left ventricular posterior wall thickness; EF, ejection fraction; FS, fractional shortening; LV Vol, left ventricular volume; d, diastole; s, systole.

| **day 28 post-MI** | **control** | **CSF2RA inh** | **p-value** |
| --- | --- | --- | --- |
| ***IVS;d (mm)*** | 0.98 ± 0.05 | 0.91 ± 0.04 | 0.3306 |
| ***IVS;s (mm)*** | 1.24 ± 0.11 | 1.24 ± 0.06 | 0.9309 |
| ***LVID;d (mm)*** | 4.22 ± 0.34 | 3.71 ± 0.12 | *0.0117 |
| ***LVID;s (mm)*** | 3.2 ± 0.31 | 2.58 ± 0.15 | *0.0126 |
| ***LVPW;d (mm)*** | 0.95 ± 0.10 | 0.83 ± 0.03 | 0.1181 |
| ***LVPW;s (mm)*** | 1.14 ± 0.12 | 1.18 ± 0.04 | 0.6476 |
| ***EF (%)*** | 48.15 ± 5.39 | 58.85 ± 3.24 | *0.0407 |
| ***FS (%)*** | 24.29 ± 2.87 | 31.12 ± 2.34 | *0.0477 |
| ***LV Vol;d (μL)*** | 80.38 ± 8.93 | 59.46 ± 4.53 | *0.0110 |
| ***LV Vol;s (μL)*** | 42.5 ± 6.21 | 25.69 ± 3.31 | *0.0150 |

**Supplementary Table S3.** Expression of Bio-Plex Pro Mouse analytes in Control (n=12) and CSF2RA inhibitor-treated mice (n=10) at 7 days post-myocardial infarction. Data represented as mean ± SEM. OOR< denotes out-of-range values, below the limit of detection. Statistical significance is reported as *P<0.05, **P<0.01, or ***P<0.001, using unpaired Students t-test.

| **Analyte** | **Control (pg/mL)** | **Csf2ra inh. (pg/mL)** | **p-value** |
| --- | --- | --- | --- |
| ***BCA-1/ CXCL13*** | 55.95 ± 8.79 | 52.32 ± 8.21 | 0.7795 |
| ***CTACK/ CCL27*** | 176.87 ± 61.08 | 337.71 ± 147.54 | 0.3201 |
| ***ENA-78/ CXCL5*** | 11.06 ± 0.28 | 15.52 ± 1.00 | ***0.0003 |
| ***Eotaxin/ CCL11*** | 58.22 ± 3.58 | 63.34 ± 3.48 | 0.3453 |
| ***Eotaxin-2/ CCL24*** | 251.03 ± 31.13 | 259.17 ± 21.04 | 0.8449 |
| ***Fractalkine/ CX3CL1*** | 8.72 ± 0.41 | 9.26 ± 0.39 | 0.3813 |
| ***GM-CSF/ CSF2*** | 0.02 ± 0.00 | 0.05 ± 0.01 | **0.0022 |
| ***I-309/ CCL1*** | 0.35 ± 0.04 | 0.66 ± 0.06 | ***0.0003 |
| ***IFN-g*** | 2.14 ± 0.73 | 1.52 ± 0.20 | 0.4781 |
| ***IL-1b*** | 1.52 ± 0.07 | 1.71 ± 0.05 | *0.0447 |
| ***IL-2*** | 0.19 ± 0.03 | 0.43 ± 0.19 | 0.1963 |
| ***IL-4*** | OOR < | OOR < | OOR < |
| ***IL-6*** | 0.55 ± 0.14 | 0.35 ± 0.04 | 0.2392 |
| ***IL-10*** | 15.73 ±1.53 | 22.46 ± 2.62 | *0.0438 |
| ***IL-16*** | 40.40 ± 5.05 | 64.30 ± 12.76 | 0.0924 |
| ***IP-10/ CXCL10*** | 67.73 ± 16.58 | 68.03 ± 15.71 | 0.9934 |
| ***I-TAC/ CXCL11*** | OOR < | OOR < | OOR < |
| ***KC/ CXCL1*** | 0.33 ± 0.07 | 0.10 ± 0.03 | 0.2770 |
| ***MCP-1/ CCL2*** | 7.84 ± 0.28 | 8.90 ± 0.33 | *0.0301 |
| ***MCP-3/ CCL7*** | 0.90 ± 0.05 | 1.22 ± 0.08 | **0.0050 |
| ***MCP-5/ CCL12*** | 0.09 ± 0.01 | 0.12 ± 0.01 | *0.0166 |
| ***MDC/ CCL22*** | 17.80 ± 1.99 | 16.55 ± 0.65 | 0.6034 |
| ***MIP-1a/ CCL3*** | 0.10 ± 0.01 | 0.19 ± 0.02 | ***0.0002 |
| ***MIP-1b/ CCL4*** | 4.37 ± 0.10 | 5.13 ± 0.12 | ***0.0002 |
| ***MIP-3a/ CCL20*** | 12.53 ± 4.97 | 7.49 ± 3.23 | 0.4594 |
| ***MIP-3b/ CCL19*** | 12.50 ± 3.14 | 14.39 ± 2.94 | 0.6838 |
| ***RANTES/ CCL5*** | 2.14 ± 0.23 | 2.40 ± 0.15 | 0.3928 |
| ***SCYB16/ CXCL16*** | 21.91 ± 4.52 | 15.35 ± 3.23 | 0.2901 |
| ***SDF-1a/ CXCL12*** | 20.85 ± 3.02 | 16.87 ± 1.30 | 0.2925 |
| ***TARC/ CCL17*** | 2.89 ± 0.11 | 3.17 ± 0.07 | 0.0573 |
| ***TNF-a*** | 4.36 ± 0.63 | 6.86 ± 0.72 | *0.0463 |

**Supplementary Table S4.** Primary antibodies used for immunohistochemistry.

| **Antibody** | **Host species** | **Stock solution** | **Dilution** | **Working concentration** | **Company** | **Catalogue number** |
| --- | --- | --- | --- | --- | --- | --- |
| **Anti-α-SMA** | mouse | 5,320 μg/mL | 1:2,000 | 2.66 μg/mL | Sigma-Aldrich | A2547 |
| **Anti-cleaved caspase 3** | rabbit | 52 μg/mL | 1:200 | 0.26 μg/mL | CST | 9661 |
| **Anti-CD68 (fluorescence)** | rabbit | 100 μg/mL | 1:300 | 0.33 μg/mL | CST | 97778 |
| **Anti-CD68 (fluorescence)** | mouse | 156 μg/mL | 1:100 | 1.56 μg/mL | Dako | M0814 |
| **Anti-CD68 (chromogenic)** | rabbit | 500 μg/mL | 1:100 | 5 μg/mL | LSBio | LS-C343891 |
| **Anti-CD206** | rabbit | 93 μg/mL | 1:300 | 0.31 μg/mL | CST | 24595 |
| **Anti-CTSZ (fluorescence & chromogenic)** | goat | 200 μg/mL | 1:100 | 2.0 μg/mL | R&D Systems | AF1033 |
| **Anti-CXCL10 (fluorescence / chromogenic)** | rabbit | 500 μg/mL | 1:75 / 1:100 | 6.67 / 5.0 μg/mL | Abcam | Ab9807 |
| **Anti-CXCR3** | rat | 500 μg/mL | 1:100 | 5.0 μg/mL | R&D Systems | MAB1685 |
| **Anti-iNOS** | rabbit | 90 μg/mL | 1:100 | 0.9 μg/mL | CST | 68186 |
| **Isolectin B4** | lectin | 1.01 mg/ml | 1:100 | 10.1 μg/mL | Sigma-Aldrich | L2140 |
| **Anti-PCNA** | mouse | 327 μg/mL | 1:100 | 3.27 μg/mL | Dako | M0879 |
| **Anti-vimentin** | rabbit | 250 μg/mL | 1:100 | 2.5 μg/mL | Abcam | Ab92547 |

**Supplementary Table S5.** Primary antibodies used for immunocytochemistry.

| **Antibody** | **Host species** | **Stock solution** | **Dilution** | **Working concentration** | **Company** | **Catalogue number** |
| --- | --- | --- | --- | --- | --- | --- |
| **Anti-α-SMA** | mouse | 44 μg/mL | 1:200 | 0.22 μg/mL | Dako | M0851 |
| **Anti-CC3** | rabbit | 200 μg/mL | 1:50 | 4 μg/mL | R&D Systems | AF835 |
| **Anti-collagen type 1** | rabbit | 500 μg/mL | 1:1,000 | 0.5 μg/mL | NSJ Bio | R31258 |
| **Anti-M-CSF-R** | rabbit | 75 μg/mL | 1:500 | 0.15 μg/mL | CST | 67455 |
| **Anti-pro-collagen type 1** | rat | - | 1:500 | - | Sigma-Aldrich | MAB1912 |

**Supplementary Table S6.** Primary antibodies used for Western blotting.

| **Antibody** | **Host species** | **Stock solution** | **Dilution** | **Working concentration** | **Company** | **Catalogue number** |
| --- | --- | --- | --- | --- | --- | --- |
| **Anti-phospho-RB (Ser807/811)** | rabbit | 240 μg/mL | 1:1,000 | 0.24 μg/mL | CST | 8516 |
| **Anti-cyclin D1** | rabbit | 78 μg/mL | 1:1,000 | 0.078 μg/mL | CST | 2978 |
| **Anti-collagen type 1** | rabbit | 500 μg/mL | 1:2,000 | 0.25 μg/mL | NSJ Bio | R31258 |
| **Anti-CTSZ** | goat | 200 μg/mL | 1:200 | 1.0 μg/mL | R&D Systems | AF934 |
| **Anti-CXCL10** | rabbit | 500 μg/mL | 1:750 | 0.67 μg/mL | Abcam | ab9807 |
| **Anti-GAPDH** | mouse | 1,000 μg/mL | 1:5,000 | 0.2 μg/mL | Millipore | MAB374 |
| **Anti-phospho-STAT5 (Tyr694)** | rabbit | 107 μg/mL | 1:500 | 0.22 μg/mL | CST | 4322 |

**Supplementary Table S7.** Primers used for qPCR.

| **Gene** | **Primer sequence** | |
| --- | --- | --- |
|  | Forward | Reverse |
| ***ACTA2*** | GGCACCCAGCACAATGAAGATCAA | TCATCTTGTTTTCTGCGCAAGTTAGG |
| ***COL1A1*** | GCTATGATGAGAAATCAACCG | TCATCTCCATTCTTTCCAGG |
| ***COL2A1*** | CAGATGTGTTTCTTCTCCTTG | GAAGAGTGGAGACTACTGG |
| ***COL3A1*** | ATTCACCTACACAGTTCTGG | TGCGTGTTCGATATTCAAAG |
| ***CCND1*** | GCCTCTAAGATGAAGGAGAC | CCATTTGCAGCAGCTC |
| ***MMP7*** | TACCCATTTGATGGGCCAGG | AGACTGCTACCATCCGTCCA |
| ***MMP12*** | TTACCCCCTTGAAATTCAGCAAGA | CGTGAACAGCAGTGAGGAACAAGT |

**
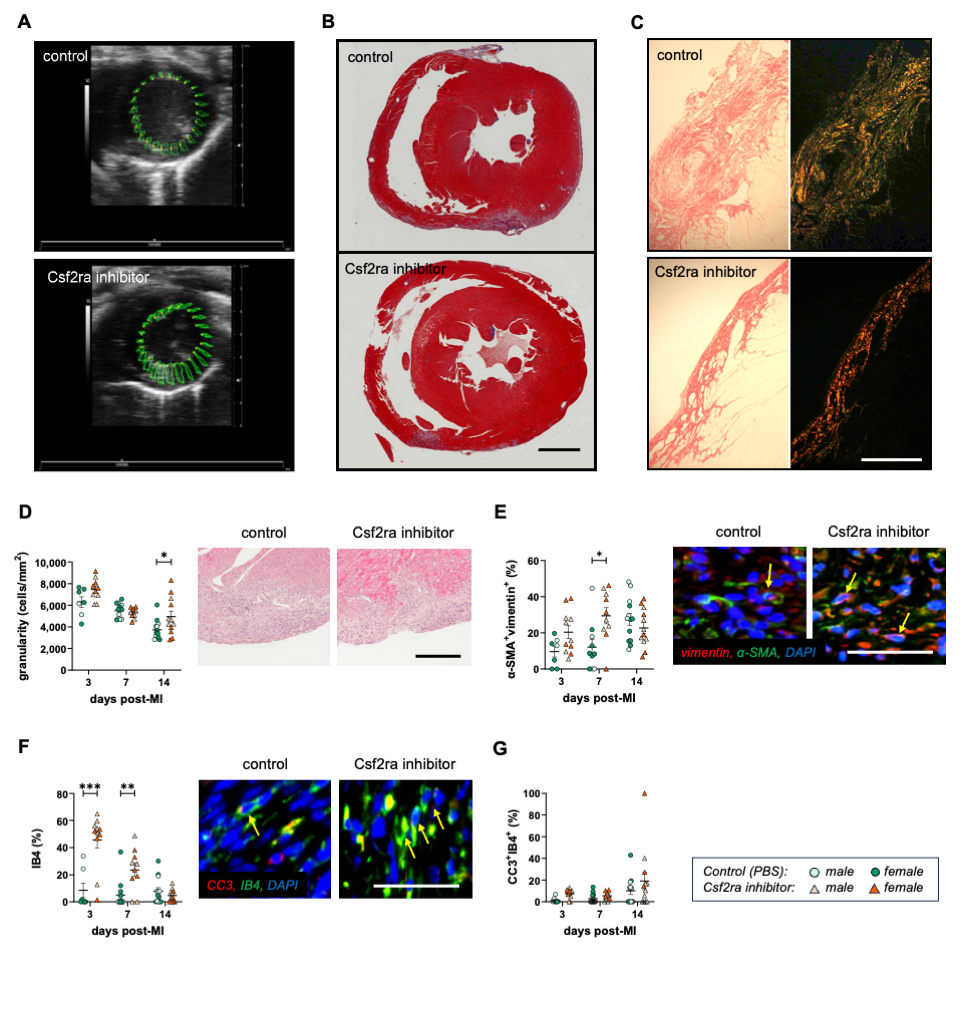
**

**Supplementary Figure S1.** CSF2RA **suppression effects on infarct granularity and cellular composition.** **(A)** Representative B-mode images demonstrating myocardial dysfunction at 3-days post-MI. **(B)** Representative cross-section images depicting fibrosis at the infarct site (blue) from Masson’s-trichrome-stained hearts in control and CSF2RA inhibitor-treated mice at 3-days post-MI. (**C**) Representative images of Picrosirius-Red-stained hearts in control and CSF2RA inhibitor-treated mice, under brightfield or linearly polarised light at 3-days post-MI. Quantification and representative images of control and CSF2RA inhibitor hearts for **(D)** granularity (cells/mm^2^), as assessed with H&E histochemistry (n=7-13); **(E)** cardiac fibroblast α-SMA expression, as assessed by dual fluorescence immunohistochemistry for α-SMA (green) and vimentin (red) (n=7-13); **(F)** capillary density and **(G)** apoptosis (n=7-13), as assessed by dual fluorescence immunohistochemistry for CC3 (red) and IB4 (green); cells positive for both markers are observed as yellow/white with examples indicated by arrows, with DAPI used as a nuclear dye. Statistical significance is reported as *P<0.05, **P<0.01 or ***P<0.001, using unpaired Students t-test. Black scale bar represents 200 μm and is applicable to panel D; white scale bar represents 500 μM and is applicable to panels E and F.


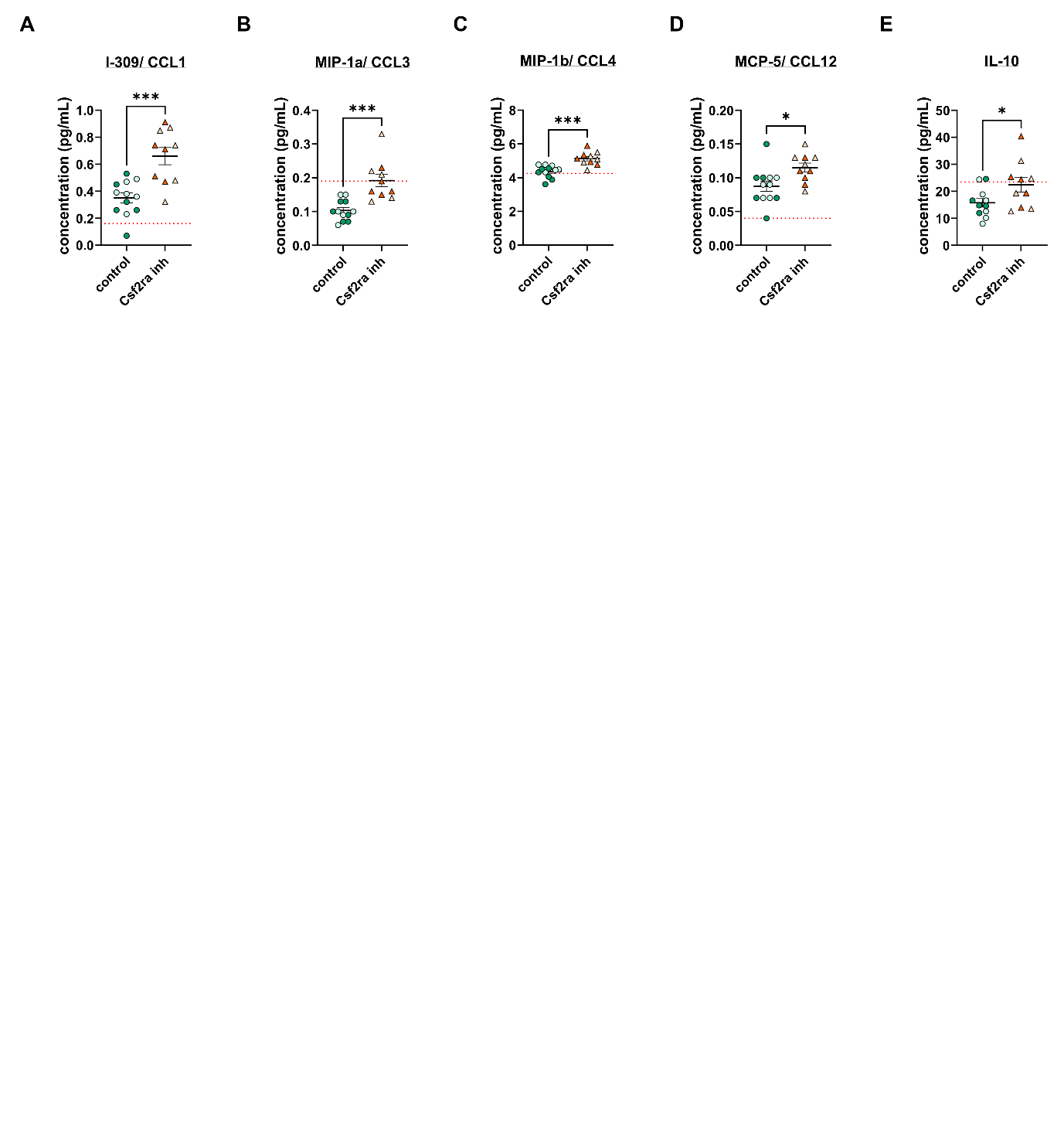


**Supplementary Figure S2.** CSF2RA **inhibition modulates chemokines/cytokines with pertinent effects on fibrotic responses.** Plasma from control (n=12) or CSF2RA inhibitor-treated mice (n=10) at day 7 post-MI was analysed using a Bio-Plex Pro Mouse Multi-Plex Assay. Quantitative graphs of select chemokines/cytokines: **(A)** CCL1; **(B)** CCL3; **(C)** CCL4; **(D)** CCL12; **(E)** IL-10. Statistical significance is reported as *P<0.05, ***P<0.001. Dashed red lines represent values from sham-operated mice (n=1).


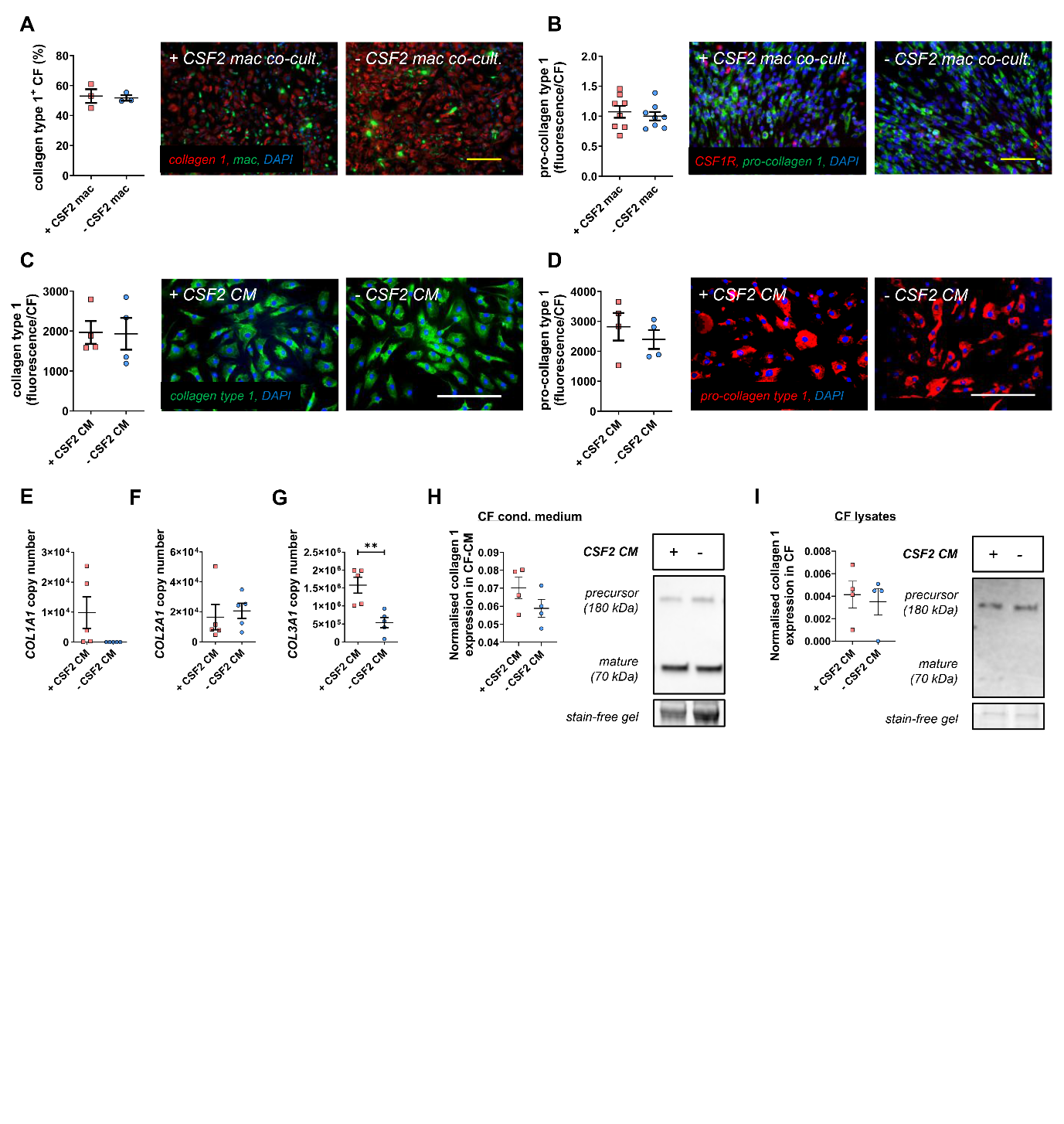


**Supplementary Figure S3. Effects of CSF2-polarised macrophage co-cultures or secretome on cardiac fibroblast collagen production.** Human cardiac fibroblasts and human primary +CSF2 or -CSF2 macrophages were co-cultured for 24 hours. Quantification and representative images of **(A)** collagen type 1 (n=3); **(B)** pro-collagen type 1 (n=8). Human cardiac fibroblasts were treated with macrophage secretome with or without CSF2 polarisation (CM) for 24 hours. Quantification and representative images of **(C)** collagen type 1 (n=4) and **(D)** pro-collagen type 1 (n=4); copy numbers of **(E)** *COL1A1*, **(F)** *COL2A1*, and **(G)** *COL3A1* (n=5); collagen type 1 protein expression in pooled cardiac fibroblast **(H)** conditioned medium (n=4) and **(I)** cell lysates (n=4)**.** Statistical significance is reported as **P<0.01. Scale bars represent 200 μm and are applicable to panels A-B (yellow) and C-D (white).


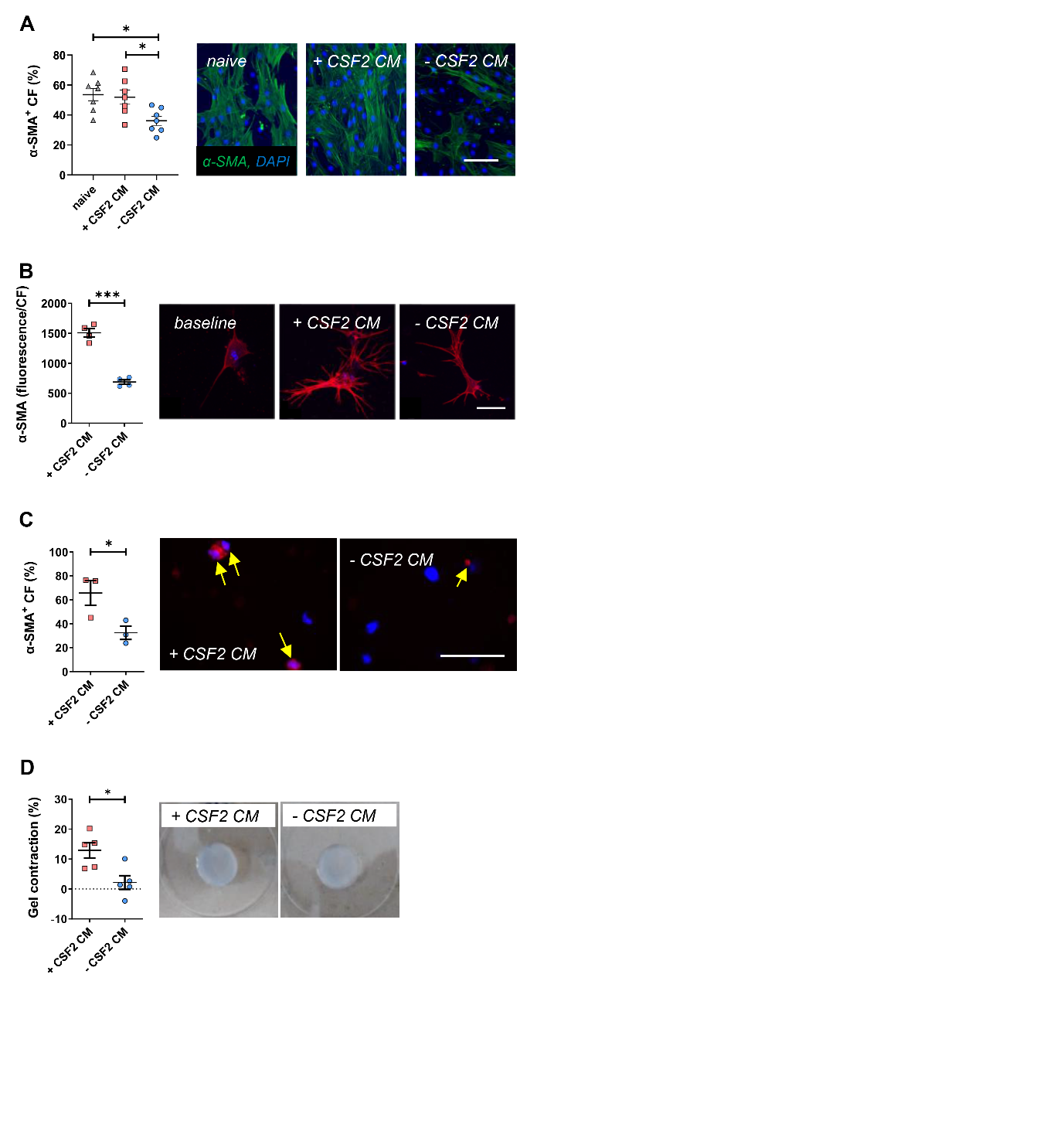


**Supplementary Figure S4. CSF2-polarised macrophages replicate the effect on cardiac fibroblast activation in low-tension environment.** Human cardiac fibroblasts were treated with macrophage secretome with or without CSF2 polarisation (CM) or remained untreated for 24 hours. Quantification and representative images of **(A)** α-SMA expression (n=7). Human cardiac fibroblasts were cultured in free-floating collagen gels for 24 hours, in order to reduce activation (baseline), and subsequently treated with +CSF2 or -CSF2 macrophage secretome (CM), and **(B)** α-SMA was assessed with confocal microscopy (n=4); **(C)** cardiac fibroblasts were extracted from the free-floating gels, centrifuged on slides and immunolabelled for α-SMA (n=3); or **(D)** cultured for 48-hours and percentage contraction calculated (n=5). Statistical significance is reported as *P<0.05 or ***P<0.001, using ordinary one-way ANOVA with Tukey’s multiple comparisons post-hoc in panel A, and paired Students t-test to panels B-D. Scale bars represent 200 μM in panel A; 50 μm in panel B; and 100 μm in panel C.


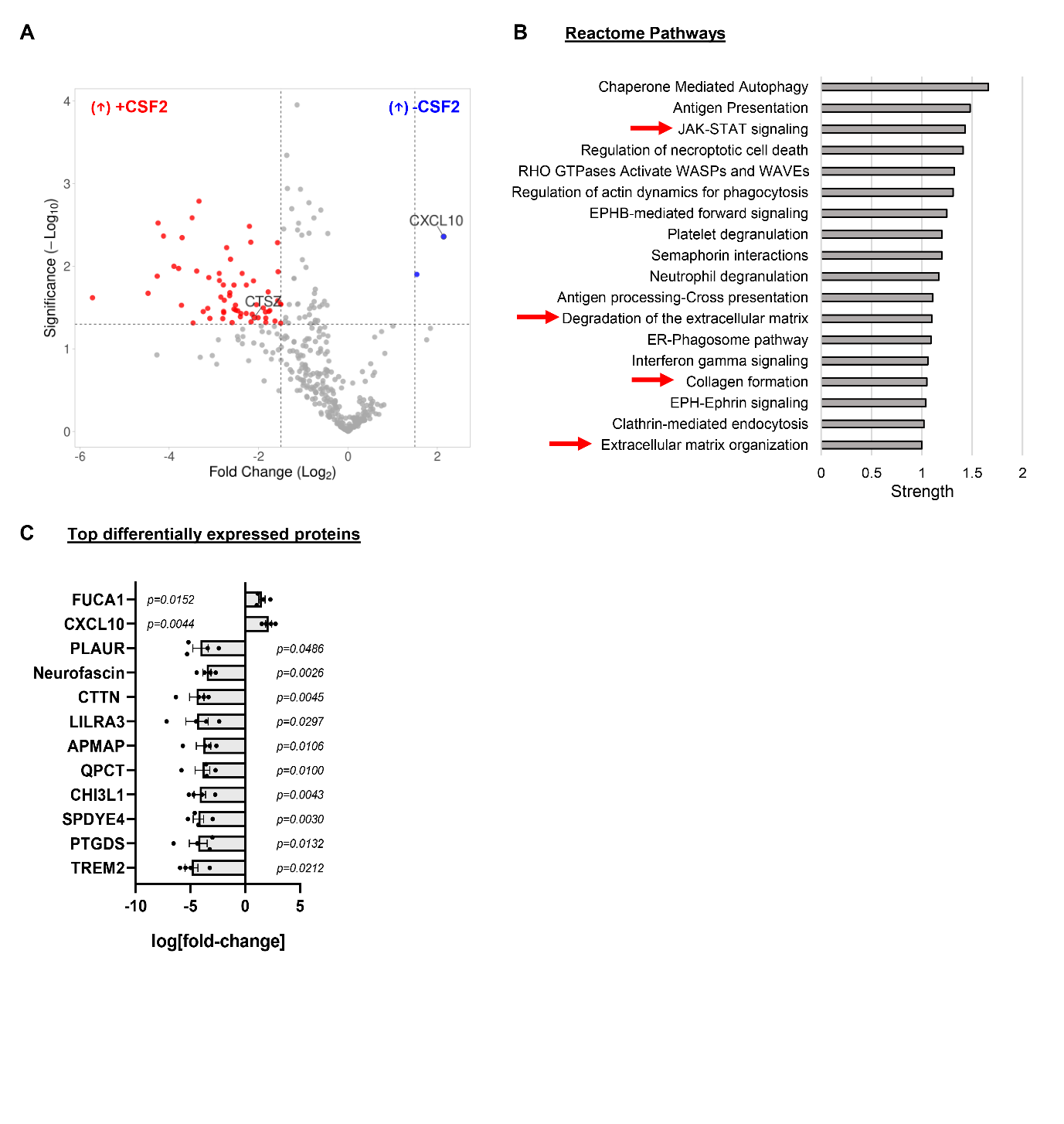


**Supplementary Figure S5. Proteomic analysis of differentially released/secreted proteins in macrophage secretome and associated bioinformatics. (A)** Volcano plot showing differentially released/secreted proteins between -CSF2 and +CSF2 macrophage secretome (n=4). Each dot represents a protein, plotted based on log[fold-change] (X-axis) and -log[p-value] (Y-axis). Grey dashed lines represent log_2_[fold-change]=1.3 and red line represents p-value=0.05. Red dots, including cathepsin Z, represent proteins that are upregulated in the +CSF2 macrophage secretome, and blue dots, including CXCL10, represent proteins that are upregulated in the -CSF2 macrophage secretome. **(B)** Identification of pathways deregulated (*P<0.05, GSEA) between -CSF2 and +CSF2 macrophages, as determined by Reactome enrichment analysis of differentially expressed proteins within respective secretomes; pertinent changed pathways are indicated by red arrows. **(C)** Top 12 differentially expressed proteins, based upon fold change.


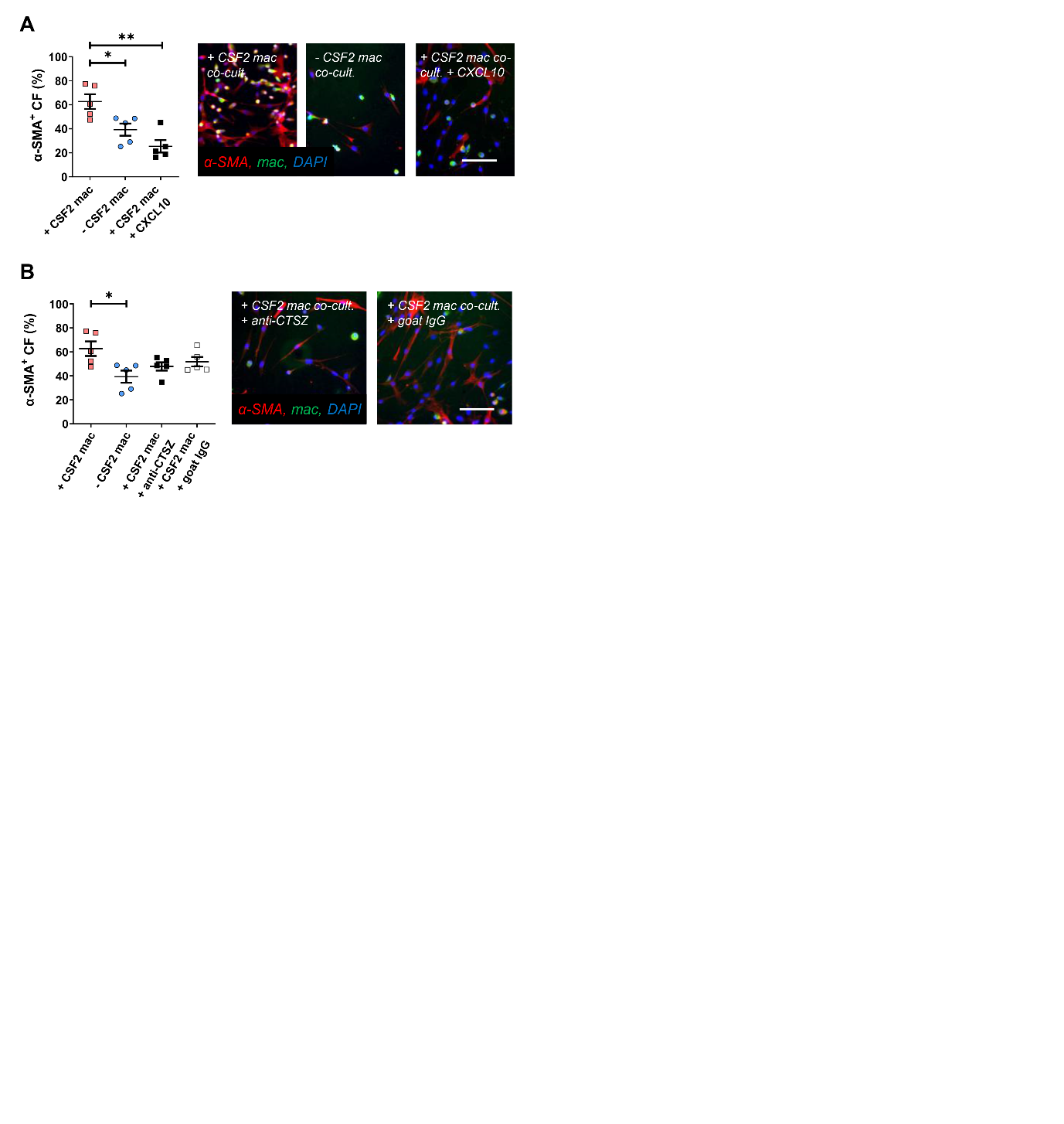


**Supplementary Figure S6. Gain-/loss-of-function experiments for CXCL10 and CTSZ in macrophage-cardiac fibroblast co-cultures.** Human cardiac fibroblasts and human primary -CSF2 or +CSF2 macrophages were co-cultured for 24 hours, alongside interventions in the +CSF2 macrophage co-cultures. **(A)** Supplementing recombinant CXCL10 (50 ng/mL) significantly reduced α-SMA expression in cardiac fibroblasts. **(B)** Inhibiting CTSZ (4 μg/mL) had a modest, but not significant reduction, in α-SMA expression, and similar effects were observed with the non-immune IgG control. Statistical significance is reported as *P<0.05 or **P<0.01, using ordinary one-way ANOVA with Tukey’s multiple comparisons post-hoc. Scale bar represents 200 μM and is applicable to all panels.


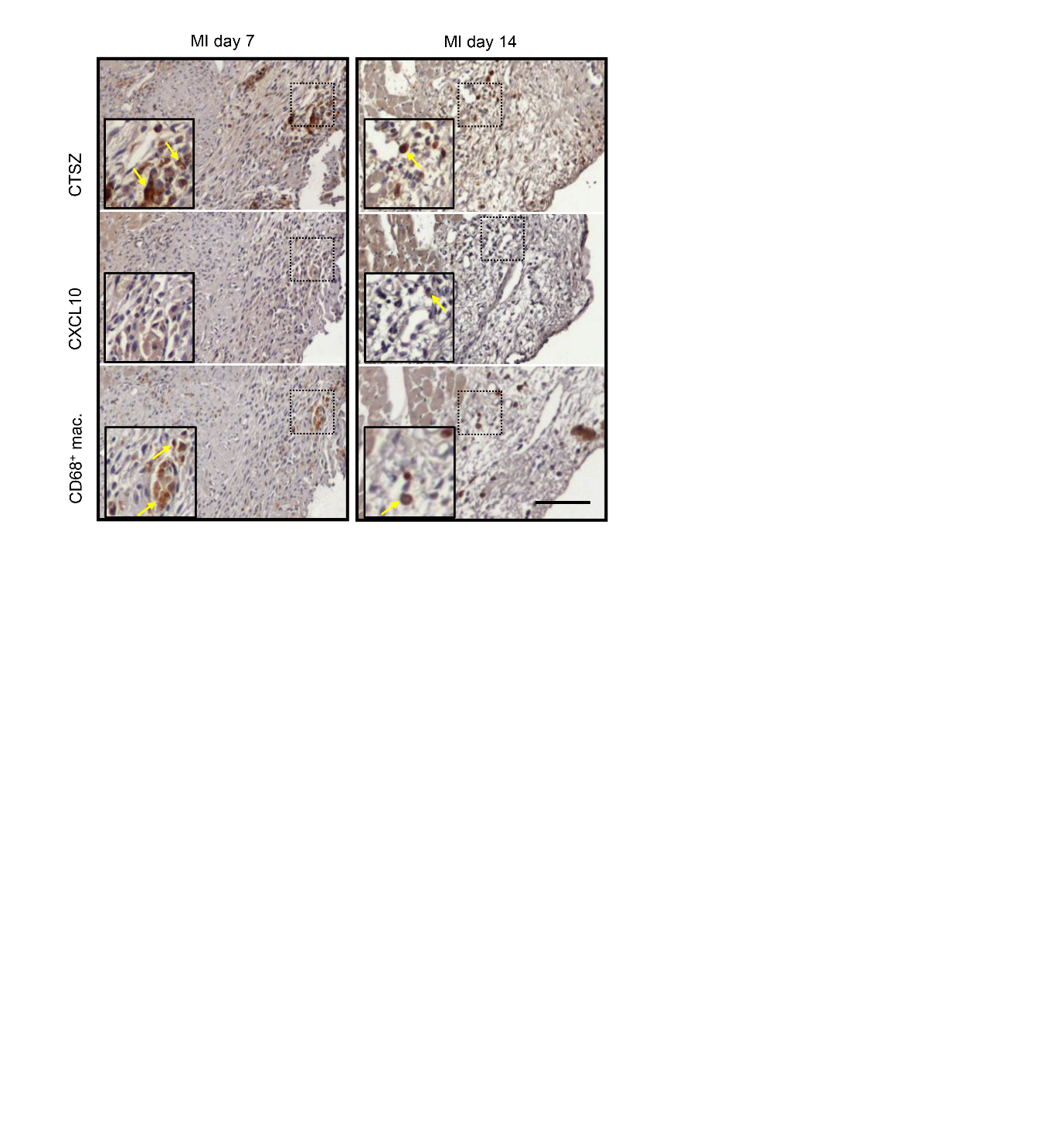


**Supplementary Figure S7. CTSZ and CXCL10 are expressed in macrophage-rich regions of the infarcts.** Representative (n=4) images of serial sections immunolabelled for CTSZ, CXCL10, and CD68. Scale bar represents 100 μM and is applicable to all panels.


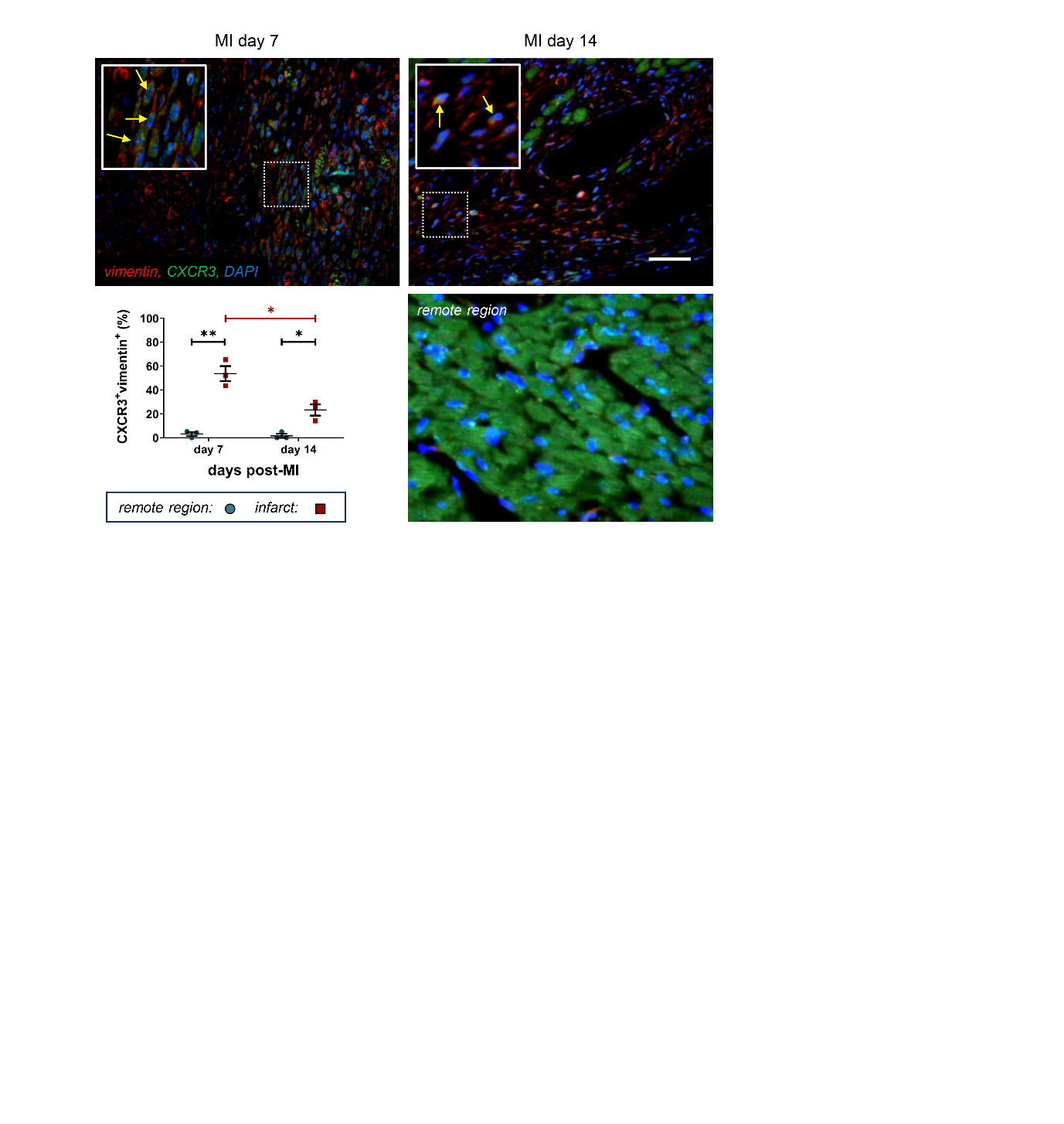


**Supplementary Figure S8. Cardiac fibroblast expression of CXCR3 at days 7 and 14 post-myocardial infarction.** Quantification and representative images of CXCR3-positive cardiac fibroblasts, as assessed in vimentin/CXCR3-stained hearts (n=3). In healthy remote heart regions, the presence of CXCR3-positive cardiac fibroblasts was minimal. Statistical significance is reported as *P<0.05 or **P<0.01, using unpaired Students t-test. Scale bar represents 500 μM and is applicable to the top panels.


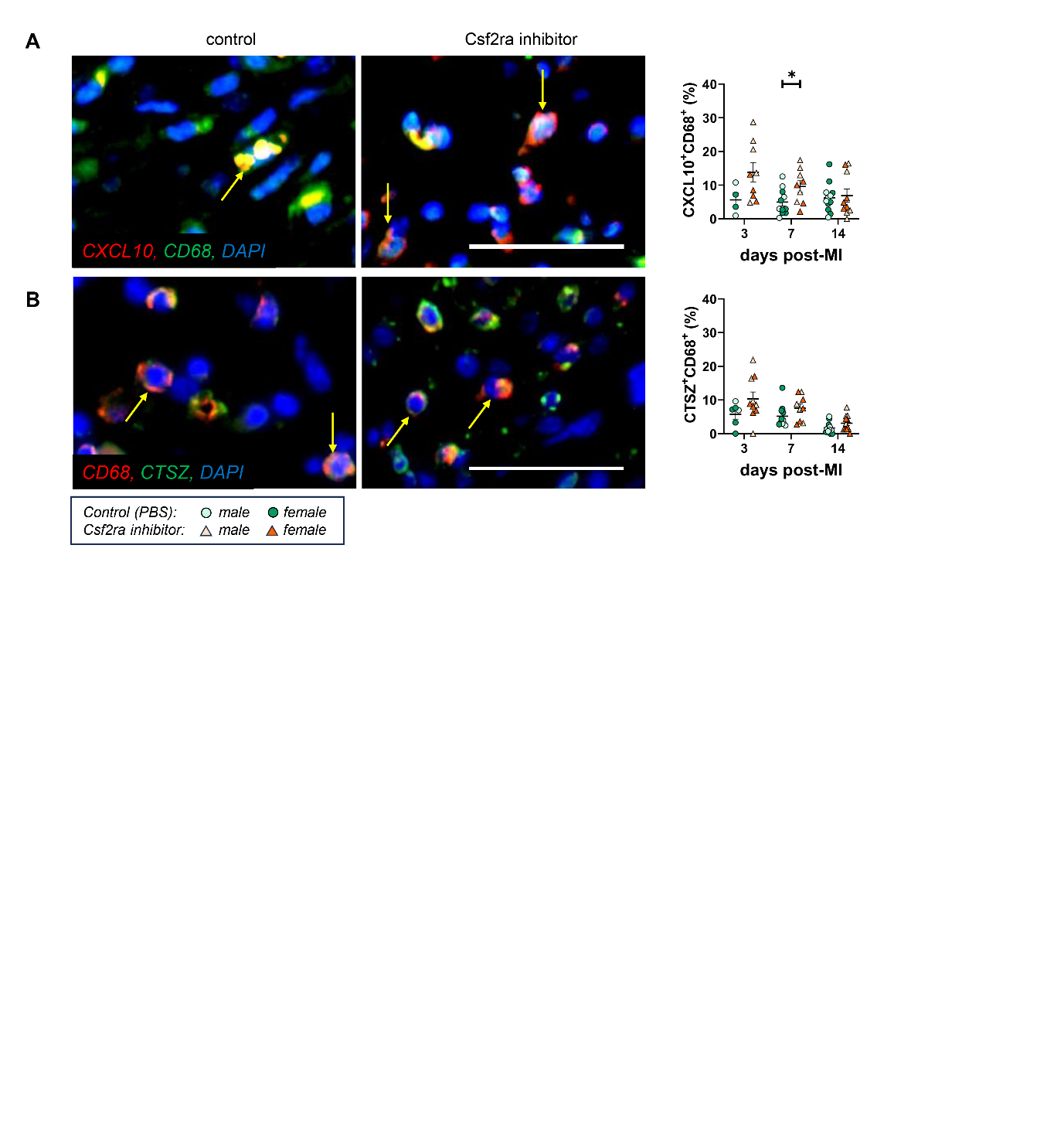


**Supplementary Figure S9.** CSF2RA **inhibition promoted CXCL10-positive macrophages, without affecting CTSZ-positive macrophages within infarcts.** Quantification and representative images of **(A)** CXCL10- (n-4-12) and **(B)** CTSZ-positive macrophages (n=6-12) in control and Csf2ra inhibitor-treated mice subjected to MI, as assessed in correspondingly immunolabelled hearts. Statistical significance is reported as *P<0.05, using unpaired Students t-test. Scale bar represents 500 μM and is applicable to all panels.


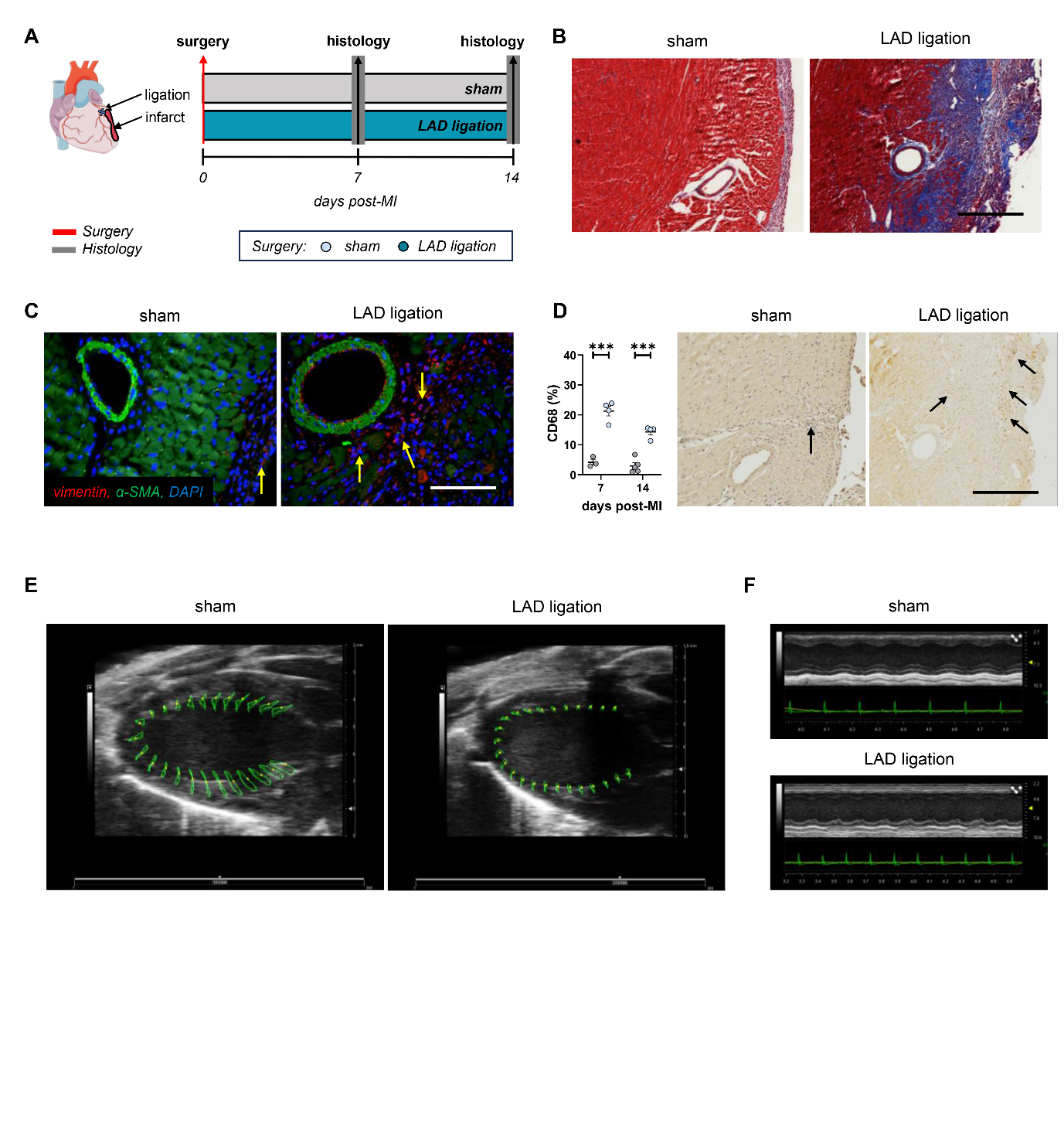


**Supplementary Figure S10. Histological validation of ischaemic injury and echocardiographic analysis in the refined myocardial infarction model of permanent LAD ligation. (A)** Experimental schematic of male C57Bl/6NCrl mice subjected to sham operation or MI through permanent LAD ligation. Representative images (day 7) of **(B)** cardiac fibrosis (μm^2^), as assessed in Masson’s-trichrome-stained hearts, and **(C)** cardiac fibroblast density and α-SMA expression, as assessed in vimentin/α-SMA-stained hearts. **(D)** Quantification and representative images of macrophage density, as assessed in CD68-stained hearts (n=3-5); Statistical significance is reported as ***P<0.001, using unpaired Students t-test. Black scale bar represents 200 μM and is applicable to panels B and D, and white scale bar represents 500 μM and is applicable to panel C. **(E)** Representative Vevo strain analysis, performed on long axis B-mode images from sham-operated and LAD ligation mice at day 14 post-surgery. **(F)** Representative M-mode images from sham-operated and LAD ligation mice at day 14 post-surgery.


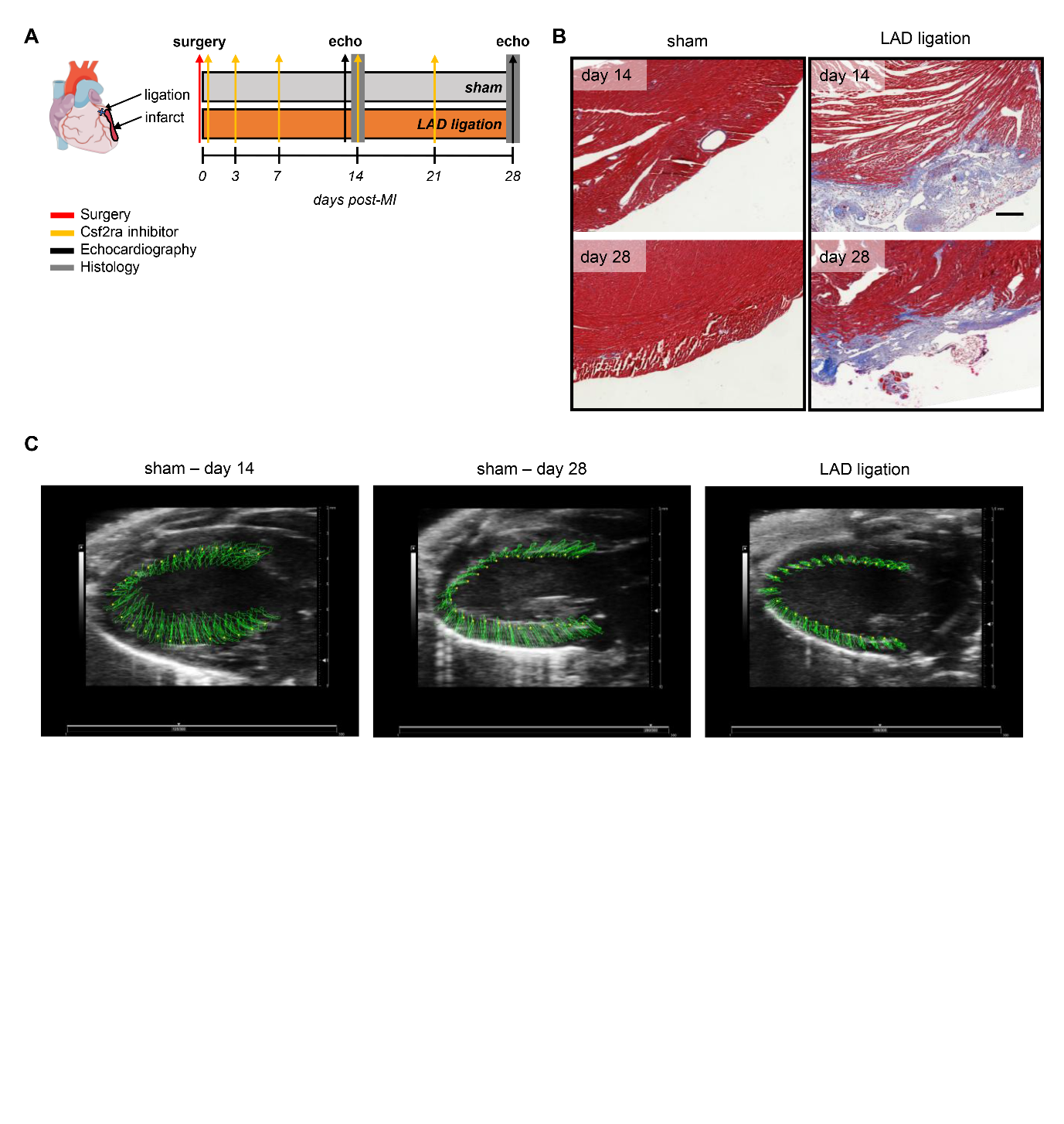


**Supplementary Figure S11. Histological validation of ischaemic injury and echocardiographic analysis in the refined myocardial infarction model of permanent LAD ligation with CSF2RA inhibition. (A)** Experimental schematic of male C57Bl/6NCrl mice subjected to sham operation or MI through permanent LAD ligation with subsequent administration of the CSF2RA inhibitor. **(B)** Representative images of cardiac fibrosis at 14 and 28 days post-surgery, as assessed in Masson’s-trichrome-stained hearts from mice that underwent sham surgery or MI and treated with the CSF2RA inhibitor, Black scale bar represents 200 μM and is applicable to panels in B. **(C)** Representative Vevo strain analysis, performed on long axis B-mode images from sham-operated (day 14 and 28) and LAD ligation mice (day 14) post-surgery.


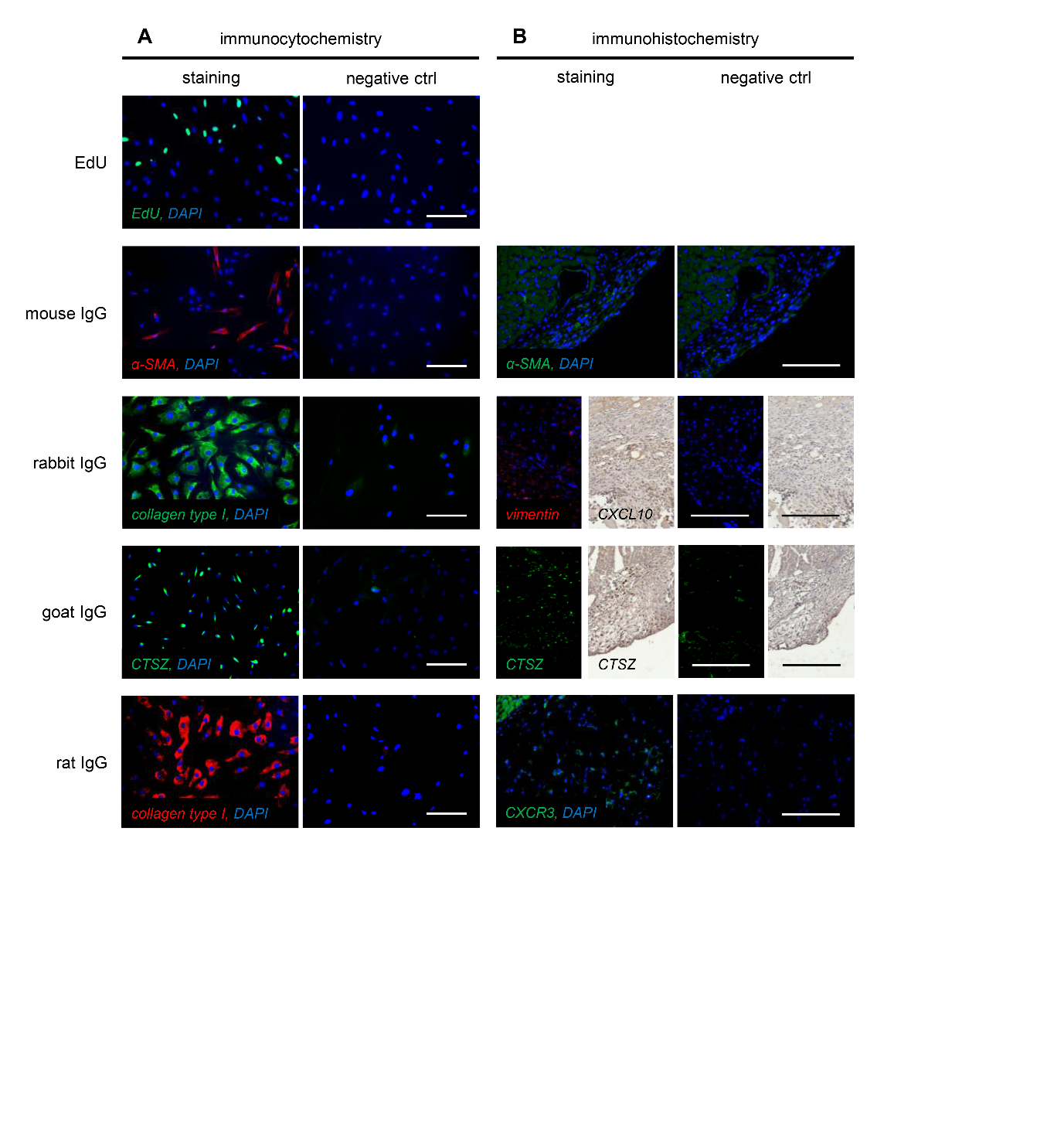


**Supplementary Figure S12. Non-immune IgG controls for immunocytochemistry and immunohistochemistry.** All immunolabelling presented was performed along with concentration- and species-matched non-immune IgG antibodies. Representative images of primary antibodies and corresponding non-immune IgG antibodies for all species are shown for **(A)** immunocytochemistry and **(B)** immunohistochemistry with chromogenic or fluorescent substrates. White scale bars in panel A represents 200 μM. Scale bars in panel B represents 500 μM.


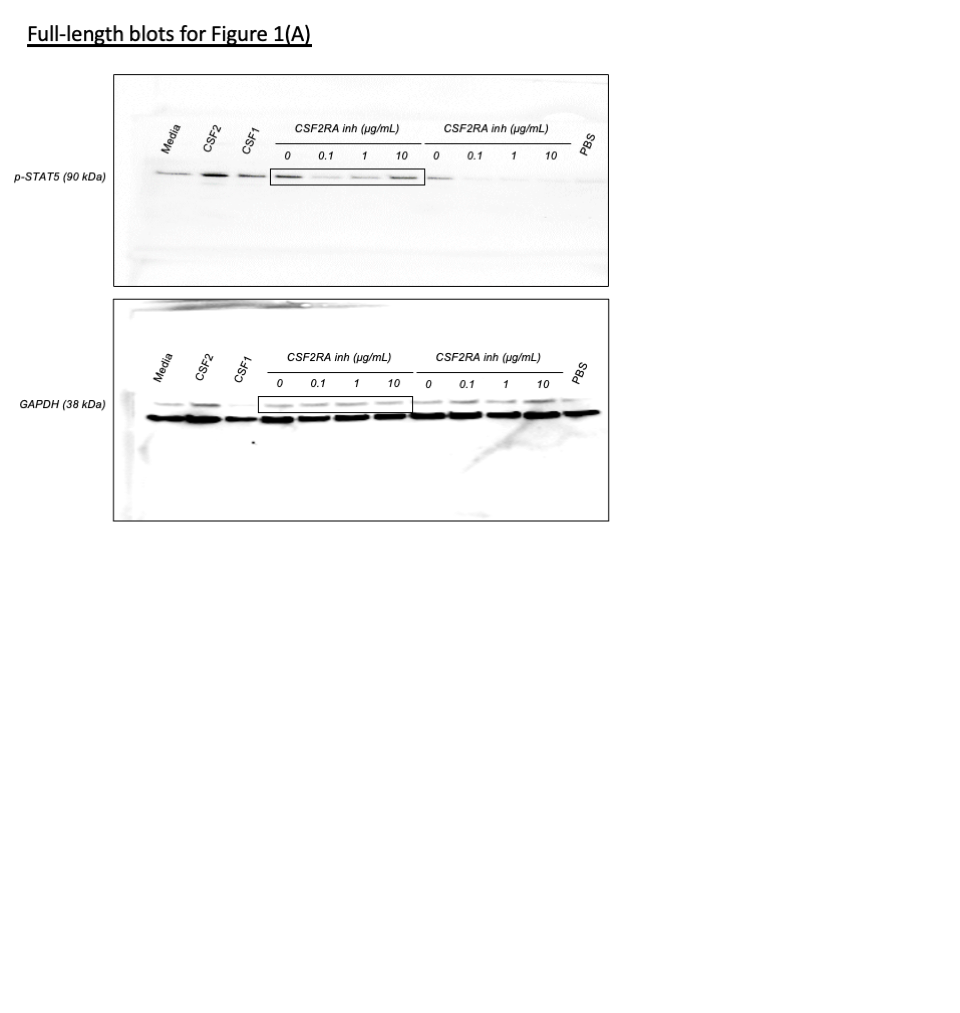


**Supplementary Figure S13. Full-length Western blots for Figure 1A.** Black boxes correspond to the cropped samples shown in the main manuscript.


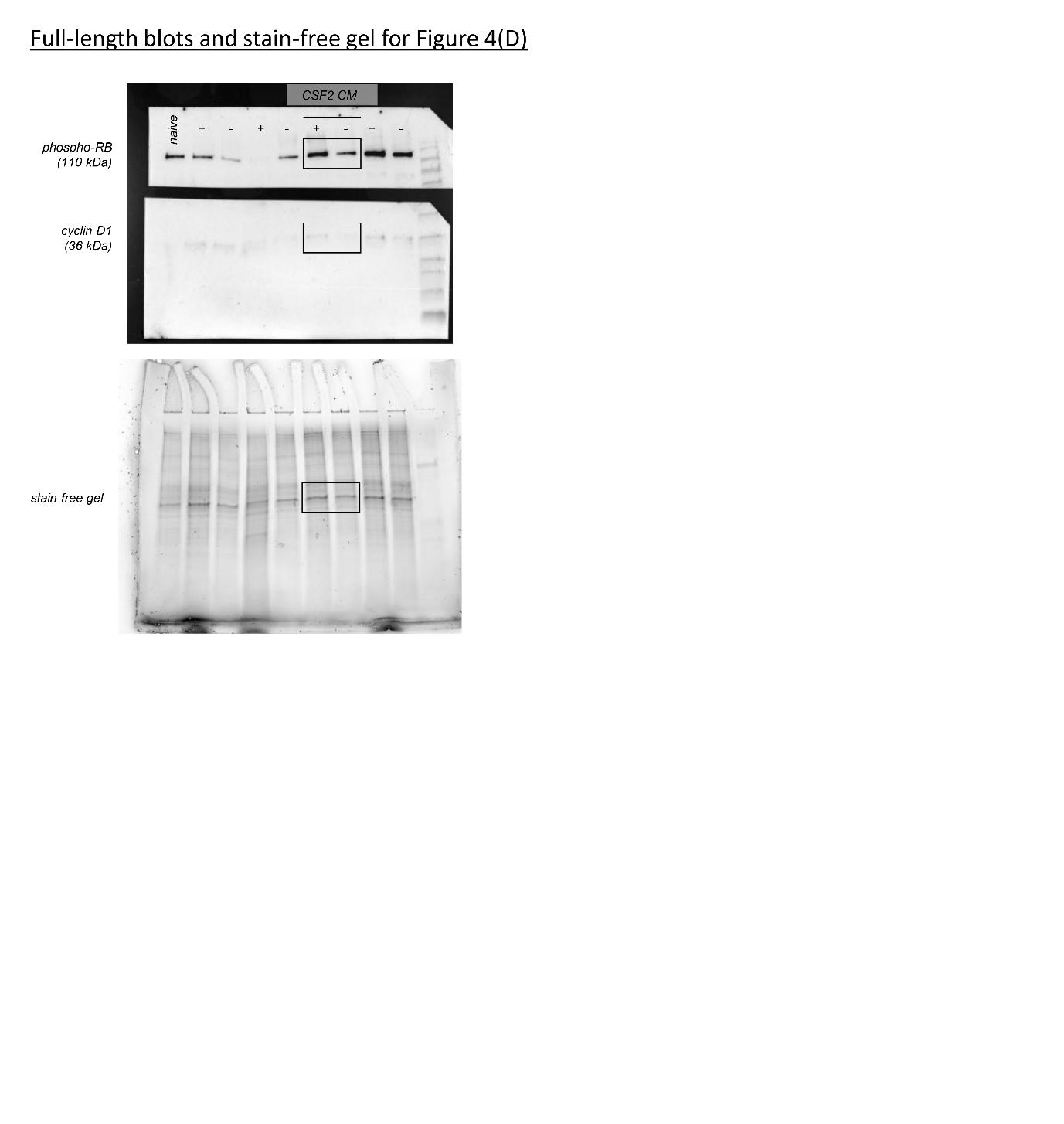


**Supplementary Figure S14. Full-length Western blots and stain-free gel for Figure 4D.** Black boxes correspond to the cropped samples shown in the main manuscript.


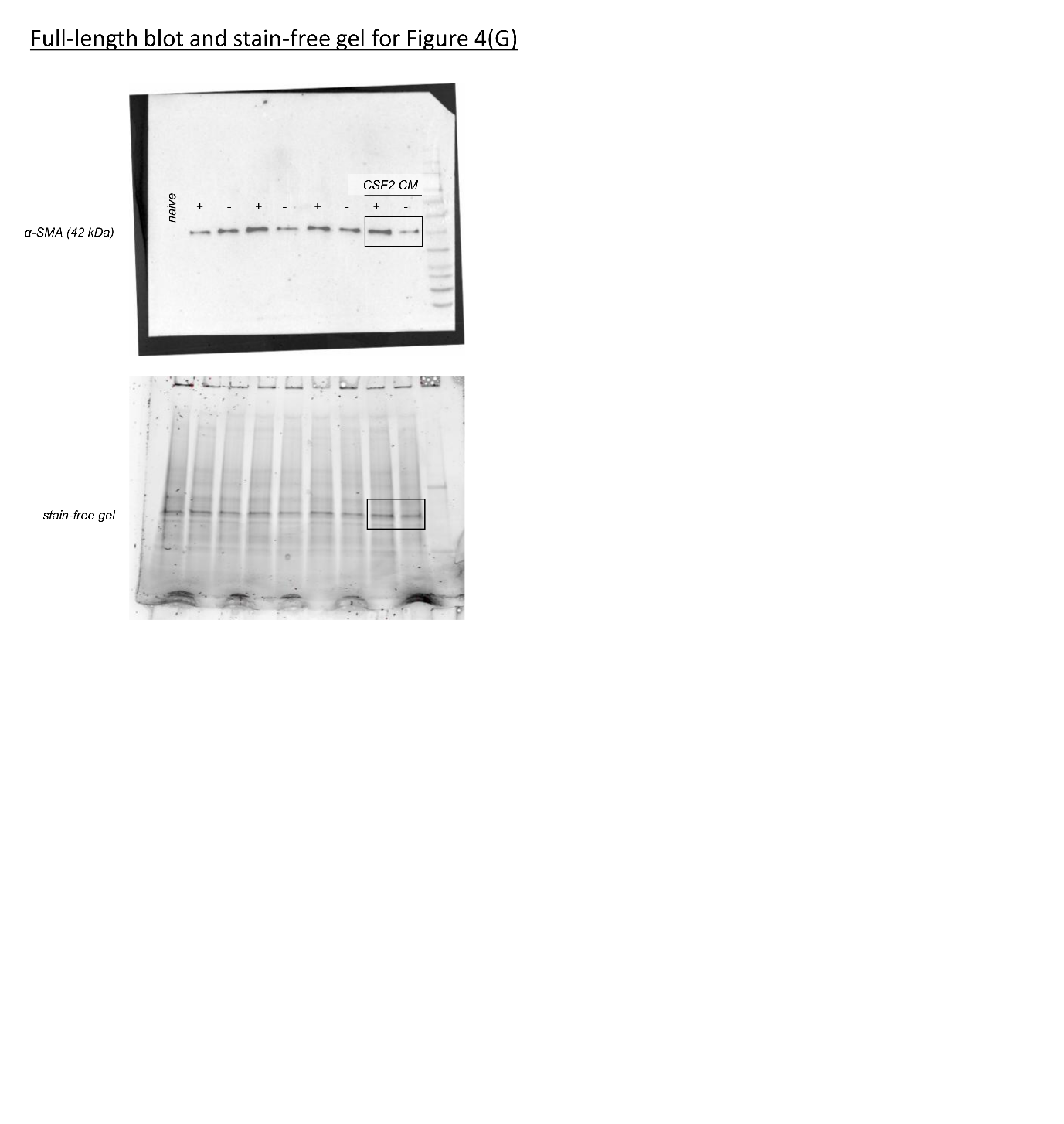


**Supplementary Figure S15. Full-length Western blot and stain-free gel for Figure 4G.** Black boxes correspond to the cropped samples shown in the main manuscript.


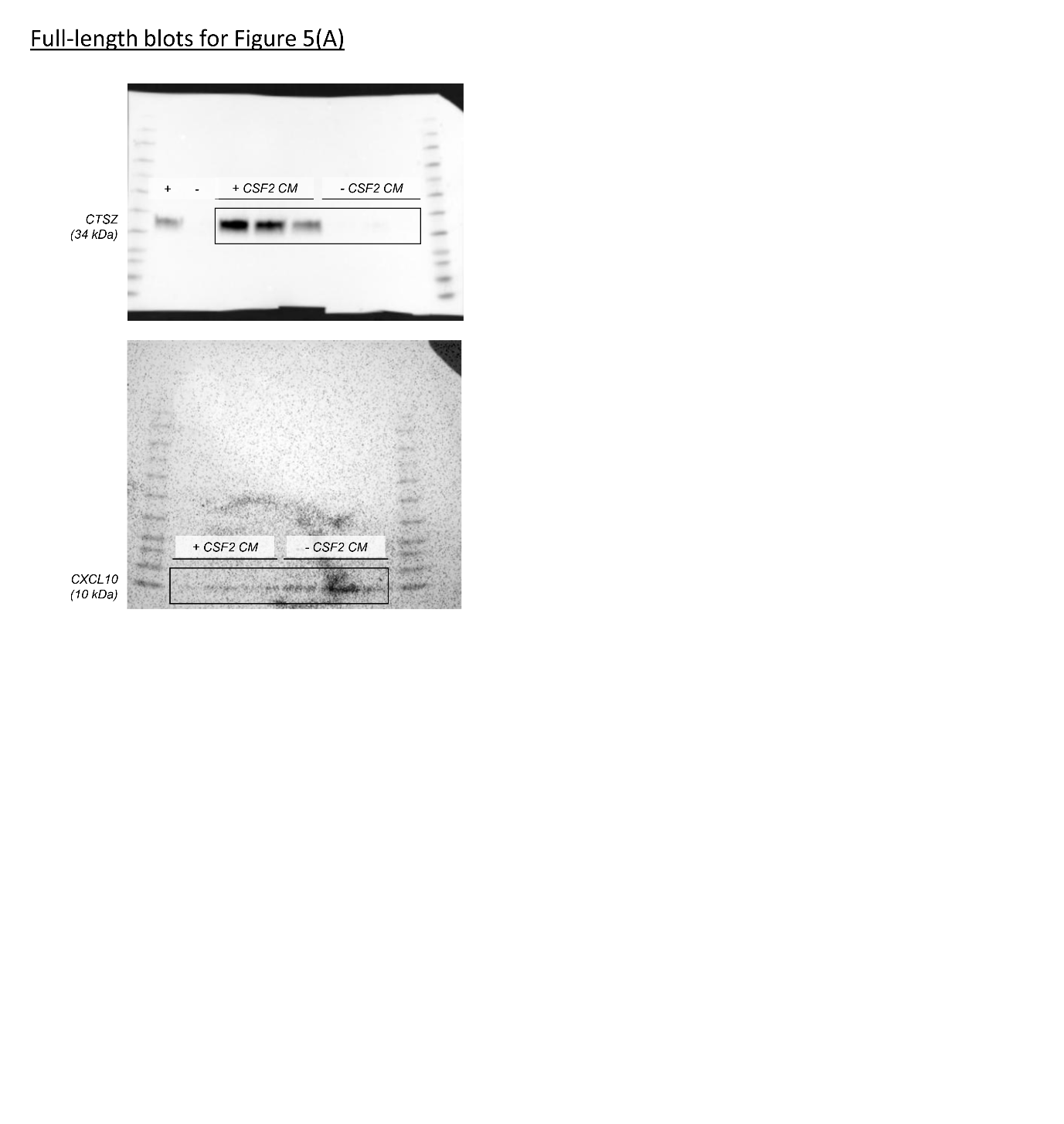


**Supplementary Figure S16. Full-length Western blots for Figure 5A.** Black boxes correspond to the cropped samples shown in the main manuscript.


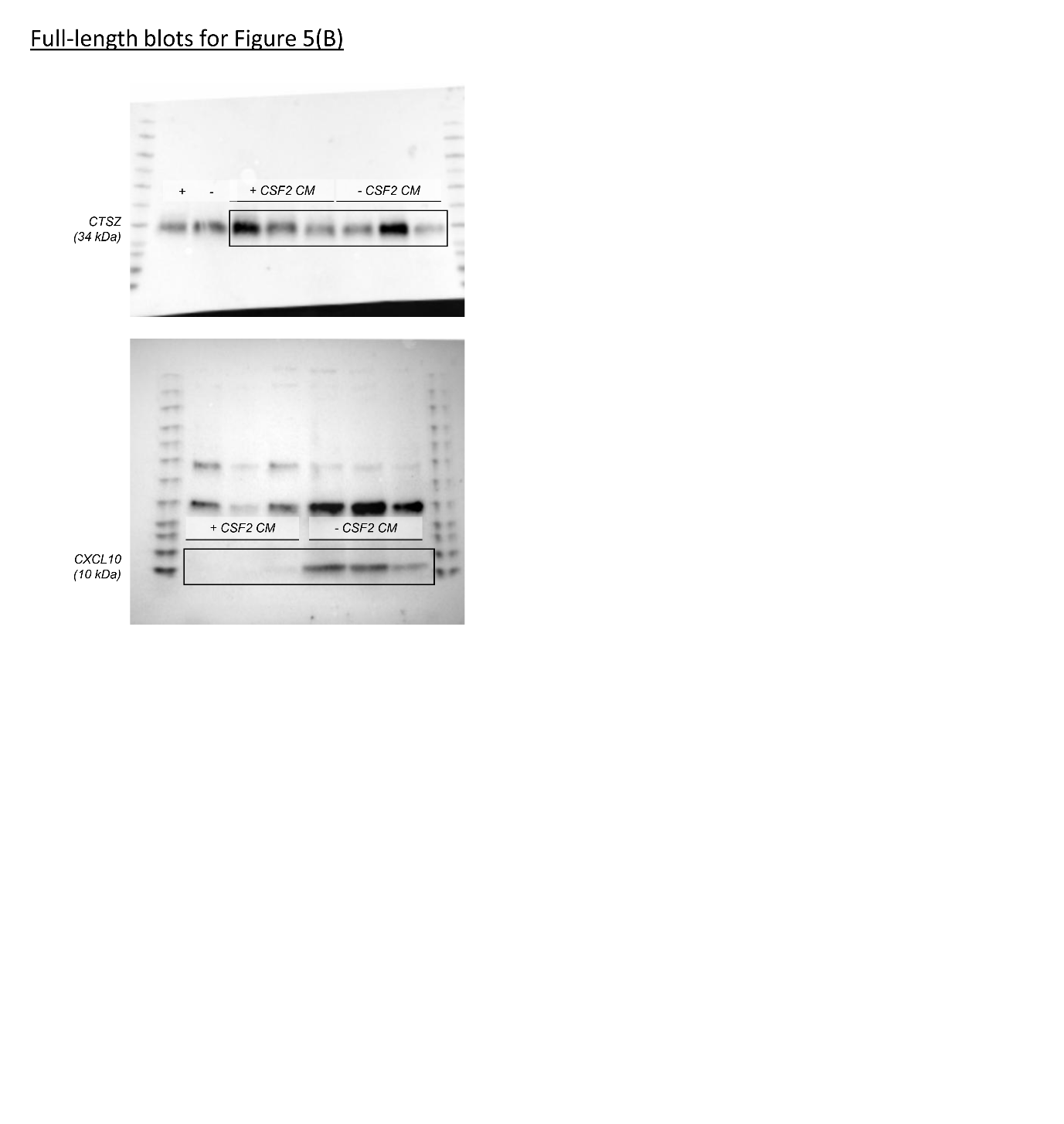


**Supplementary Figure S17. Full-length Western blots for Figure 5B.** Black boxes correspond to the cropped samples shown in the main manuscript.


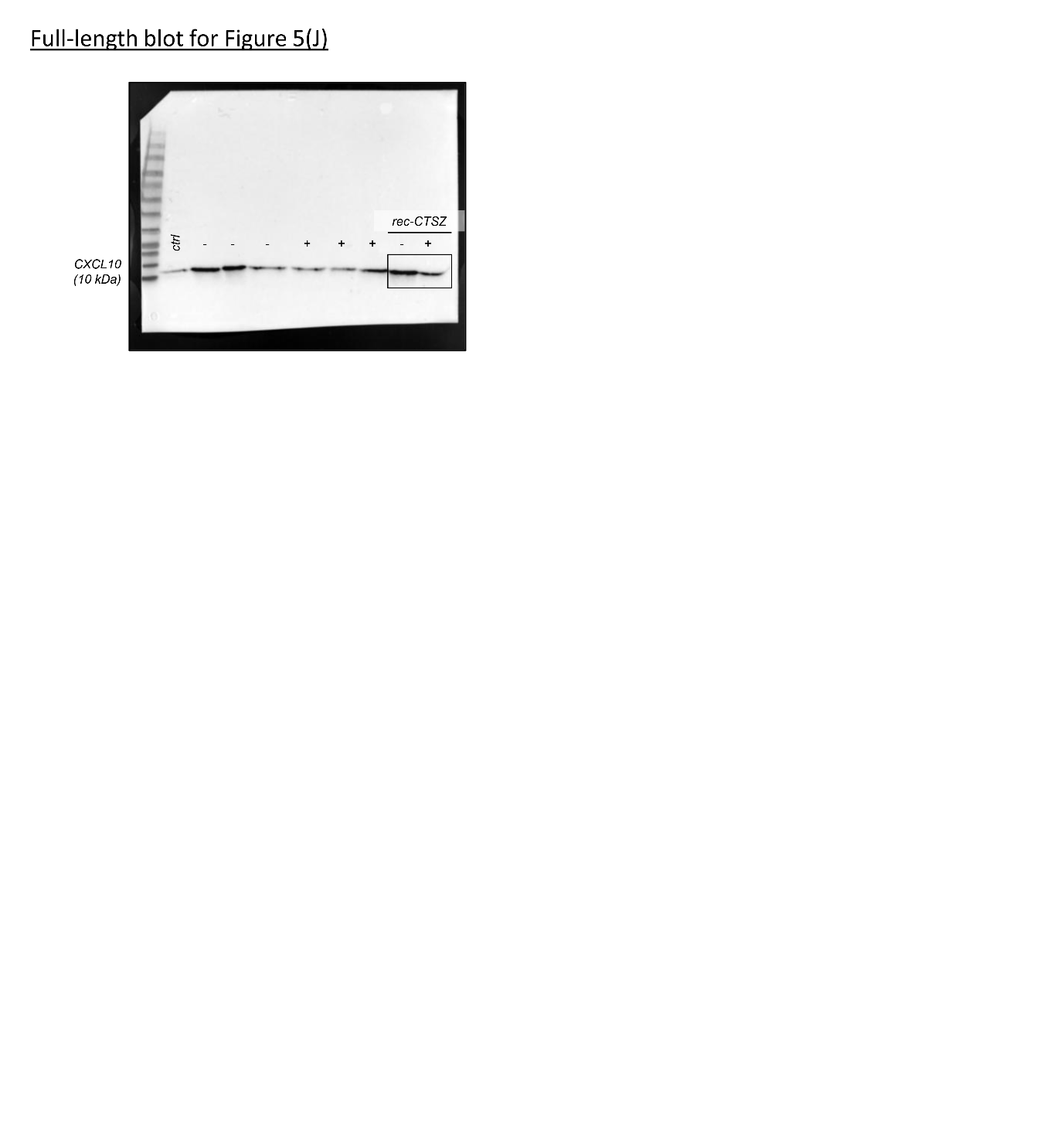


**Supplementary Figure S18. Full-length Western blot for Figure 5J.** Black boxes correspond to the cropped samples shown in the main manuscript.


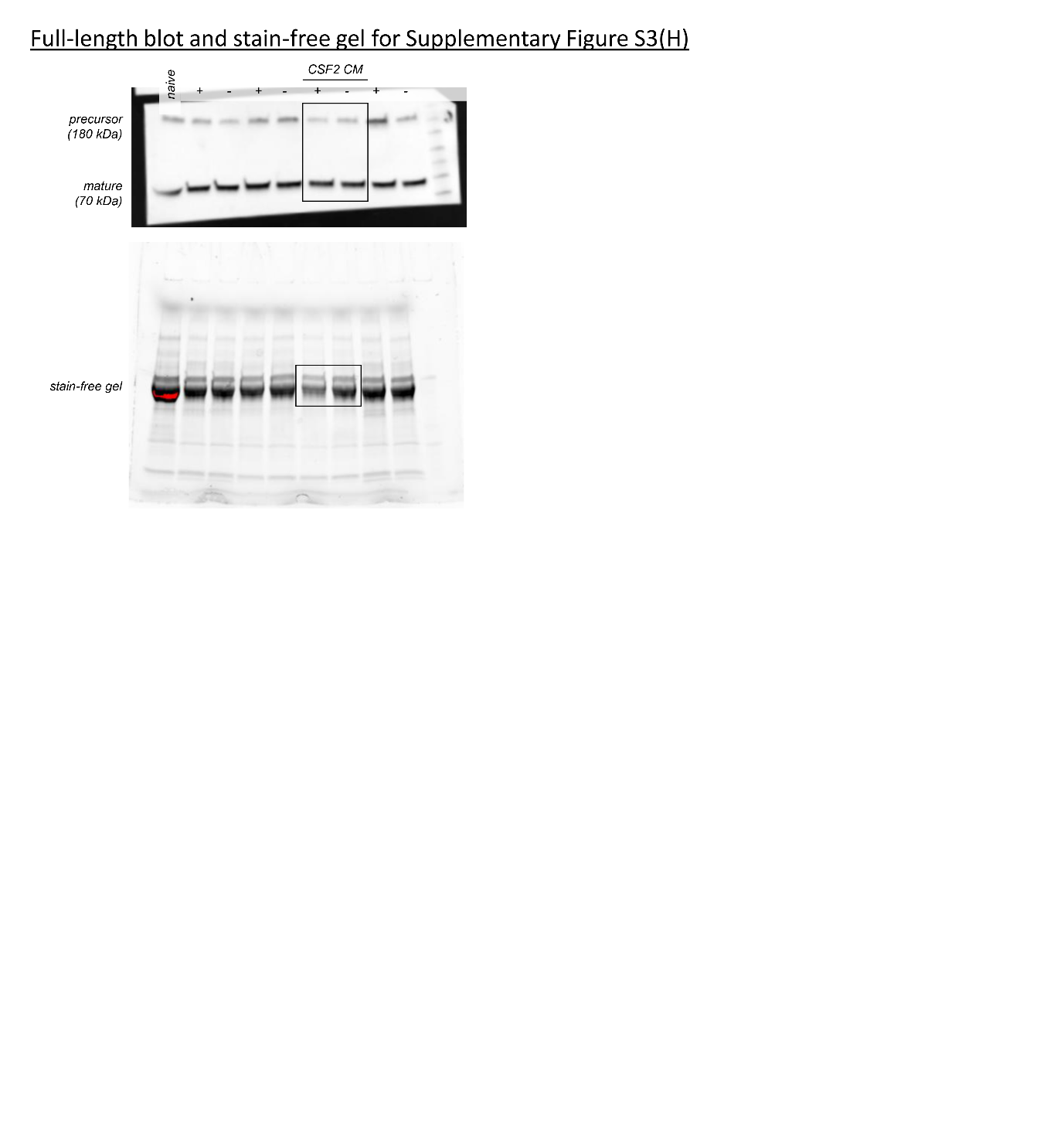


**Supplementary Figure S19. Full-length Western blot and stain-free gel for Supplementary Figure S3(H).** Black boxes correspond to the cropped samples shown in the main manuscript.


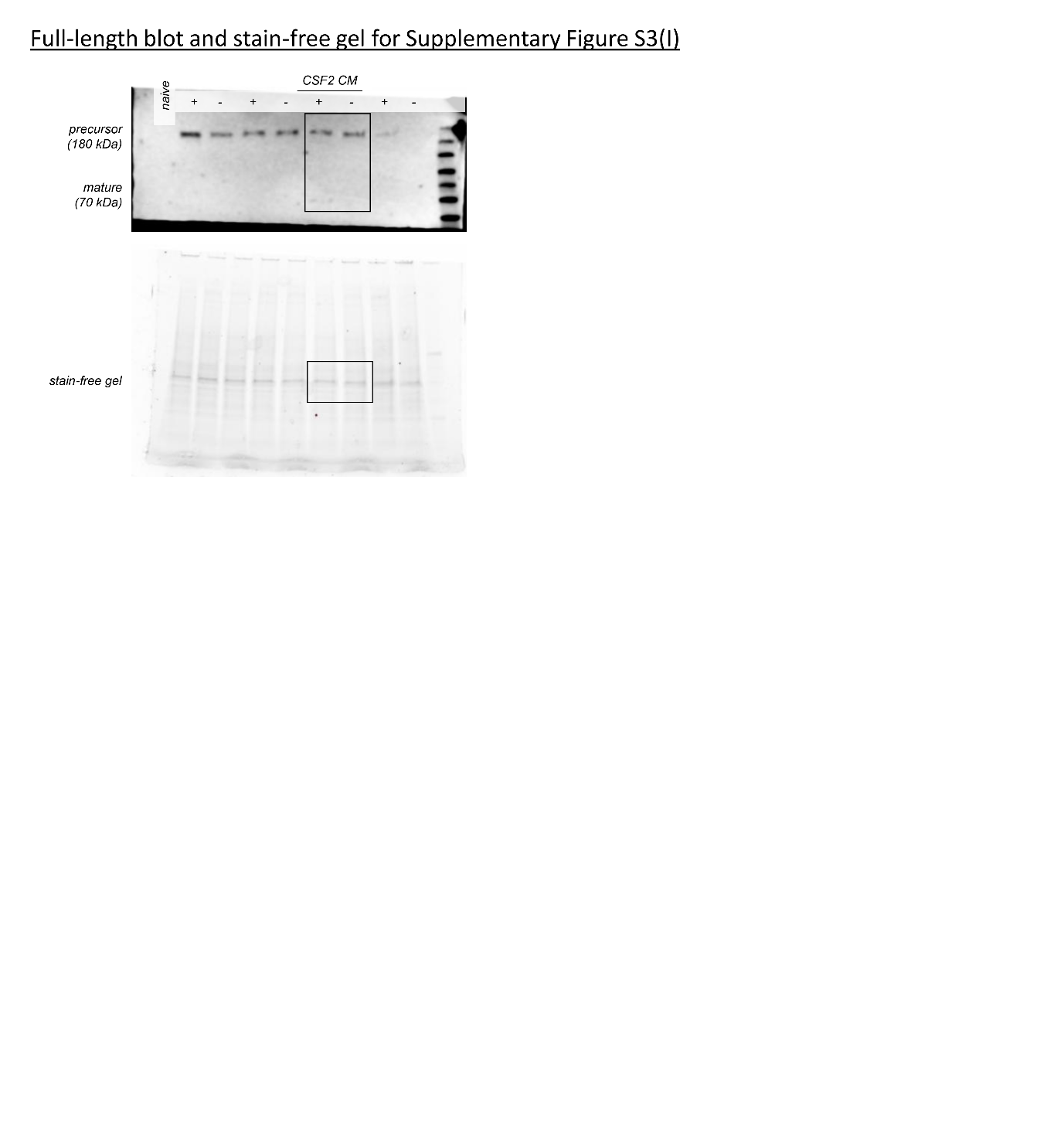


**Supplementary Figure S20. Full-length Western blot and stain-free gel for Supplementary Figure S3(I).** Black boxes correspond to the cropped samples shown in the main manuscript.
